# Systematic Evaluation of a Cluster-Continuum-Model Workflow to Compute the Free Energies of Solvation of Ions in Different Solvents

**DOI:** 10.1021/acs.jpca.6c00886

**Published:** 2026-04-28

**Authors:** Morten Lehmann, Froze Jameel, Martin Kaupp

**Affiliations:** Technische Universität Berlin, Institut für Chemie, D-10623 Berlin, Germany

## Abstract

A partially automatized computational protocol for the construction of embedded microsolvated clusters based on a combination of the quantum cluster growth algorithm, DFT reoptimization, higher-level energy computations, conformer selection based on free energy, embedding into advanced implicit solvation models like COSMO-RS, and averaging over a range of cluster sizes has been validated and applied to calculate single-ion Gibbs free energies of solvation in different solvents. For aqueous solution, excellent agreement with a widely accepted single-ion scale based on the cluster pair approximation (CPA) is found, to within the error margins of that scale, but without anchoring to one particular ion like the latter. Similar calculations show that the TATB assumption underlying the second widely used single-ion scale is not justified, shifting the TATB scale even further from the CPA scale. Applications of the cluster-continuum method to single-ion values in acetonitrile show that here the CPA-based proton value may be less settled than with water, methanol, or DMSO, and it deserves closer examination. Finally, the solvation of the fluoride ion is compared in a variety of different solvents (water, acetonitrile, methanol, DMSO, diethyl ether, and benzene). While water and methanol provide the most negative solvation free energies, acetonitrile, methanol, and DMSO are not far behind, and even the relatively nonpolar diethyl ether and benzene exhibit appreciable stabilization. This is due to significant charge-assisted C–H···F hydrogen bonds to the highly compact fluoride ion in all of the formally aprotic solvents, involving in some cases more than one proton from a given solvent molecule in a chelate binding mode.

## Graphical abstract

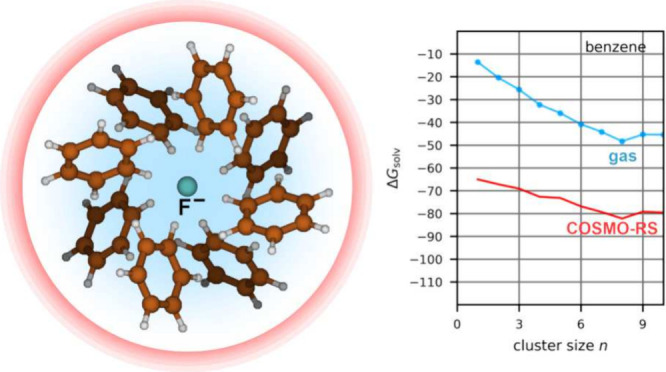

## 1 Introduction

Most chemical reactions are carried out in solution. If we want to model them quantum-chemically, the solvent should therefore be considered in some way. While in the early days of quantum chemistry typical calculations referred to the gas phase at 0 K, the inclusion of solvent effects in such calculations has become a major field of research over the past decades. The methods to model the solution environment cover a wide range of sophistication and computational complexity, which has to be tailored to the chemical questions at hand and to the available manpower, time, and computational resources. A detailed exposition of these various methodologies goes beyond this introduction, and we refer the reader, e.g., to refs 1–3 and references cited therein. In Figure 1 we show a rough classification of complexity levels. At the low end of the sophistication/complexity scale we find polarizable (dielectric) continuum models (PCMs),^4,5^ of which the conductor-like screening model (COSMO)^6^ is a prominent example. At the upper end of the scale we find explicit *ab initio* molecular dynamics (AIMD) simulations of the entire solute–solvent system with subsequent thermodynamical integration,^7,8^ which covers all relevant interactions but can be prohibitive in terms of computational and human resources for many routine applications. Simplification of AIMD simulations by treating only the solute quantum-mechanically (QM) but the solvent only at the molecular-mechanics (MM) level in these MD simulations provides the QM/MM level. We mention also more computationally expedient hybrid models like the three-dimensional reference interaction site model (3D-RISM-SCF)^9–12^ or other mean-field treatments of solvation that may be viewed to provide the result of approximately averaging over a QM/MM MD simulation (Figure 1). PCMs cover only the bulk electrostatic contributions of solvation and typically miss specific solvent–solute contributions, but there have been semiempirical extensions. Two important models we will use in this work are the SMD model (Solvation Model based on Density^13^) that adds semiempirical contributions to a PCM framework, and in particular the “conductor-like screening model for real solvents” (COSMO-RS),^14^ that starts from COSMO but then uses statistical thermodynamics in an integral equation formalism and adds also semiempirical contributions, in particular to cover hydrogen bonding.

**Figure 1:**
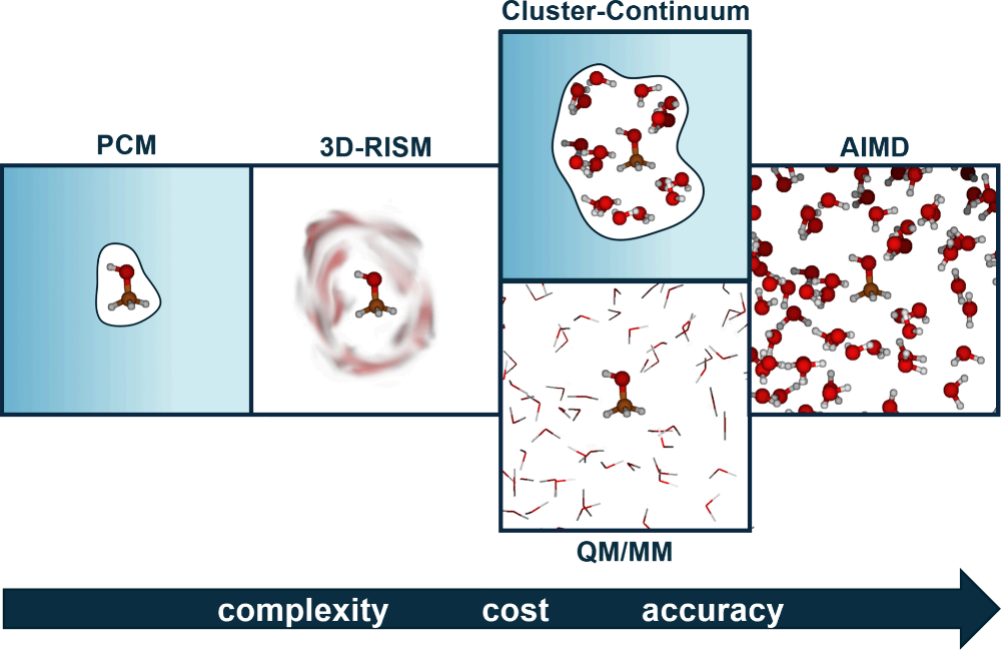
Schematic overview of an incomplete hierarchy of solvation models. 3D-RISM-SCF may be viewed as a simplified QM/MM treatment, where the time average over a QM/MM MD simulation is replaced by the averaged (smeared-out) MM solvent distribution of an integral equation.

Parameterized models like COSMO-RS or 3D-RISM-SCF can go a long way toward bringing in more specific interactions at affordable cost and COSMO-RS has been, in particular, very successful in modeling solvation free energies even in the presence of important hydrogen-bonding interactions. However, there are situations where a quantum-mechanical treatment of solvent molecules may be unavoidable, but where a full AIMD simulation of the solute–solvent system with thermodynamic integration is too demanding. As just one representative example of questions where a quantum-mechanical treatment of the solvent is essential, we mention here strong electronic couplings between solute and solvent orbitals in the computation of response properties like the NMR chemical shifts of solute nuclei hydrogen-bonded to the solvent.^15,16^ This is just one of the areas where cluster-continuum models are of importance and have become popular.^17–27^ That is, one includes a limited number of solvent molecules in the close environment of the solute quantum-mechanically but then embeds the resulting cluster into an implicit solvent model like COSMO, SMD, or COSMO-RS. Central questions for the application of such models are a) the necessary size of the QM part, i.e., of the solute–solvent cluster, b) how to account for conformational selection and possibly averaging of the dynamical solvent environment in a computationally expedient way, while getting accurate binding energies and polarization contributions, and c) what is the effect of the implicit model used for the embedding.

In this work we carry out a systematic evaluation of automated protocols for the construction of microsolvated clusters of solvated ions, including aspects of conformational selection and averaging, cluster size, electronic-structure method, and embedding model. As we will focus on the wide and challenging field of single-ion Gibbs free energies of solvation, the difficult question of the choice of single-ion solvation scales arises. Cluster construction will be based on the so-called Quantum Cluster Growth (QCG) algorithm reported recently for an automated setup of microsolvated clusters^28^ allowing the computationally expedient construction and conformational selection and/or averaging of conformers, using the classical force-field (GFN-FF^29^) or tight-binding semiempirical MO levels (GFN2-xTB^30^) of the CREST program.^31,32^ Structures and energies are then refined at different density functional theory (DFT) levels. Among the many other methods suggested for the automatic construction of microsolvated cluster models,^33–37^ we will also have a brief look at the SOLVATOR/GOAT algorithm in ORCA.^38^ Combination of these models allows larger cluster sizes to be evaluated than usually done in this field, while still providing the required low-lying conformers, which permits a more detailed examination of convergence of free energy of solvation with cluster size. While we will start by evaluating single-ion free-energy scales in aqueous solution, a main goal of this work is to look at nonaqueous solvents. In particular, we will examine charge-assisted hydrogen bonding to fluoride^15^ in different solvents.

## 2 Theoretical Background

### 2.1 Cluster-Continuum Approach

To calculate free energies of solvation with a cluster-continuum approach, we formulate a thermodynamic cluster cycle (see Figure 2),^25^ leading to the following expression for the solvation free energy:

1 $$\Delta G_{solv}^{*}\left(A^{m\pm }\right)=\Delta G_{g,bind}^{{}^{\circ}}+\Delta G_{solv}^{*}\left({\left[{A\left(S\right)}_{n}\right]}^{m\pm }\right)-\Delta G_{solv}^{*}\left({\left(S\right)}_{n}\right)-\Delta G^{{}^{\circ}\to *}+\Delta G_{{\left(S\right)}_{n}}^{l\to *}$$

$\Delta G_{g,bind}^{{}^{\circ}}$ is the gas-phase binding energy of an arbitrary solute A^*m*±^ with a solvent cluster (S)_*n*_ to form the solute–solvent cluster ${\left[{A\left(S\right)}_{n}\right]}^{m\pm }$ . $\Delta G_{solv}^{*}$ are the Gibbs free energies of solvation. It has been pointed out^25^ that it is important to take care of the different standard states. While the quantum-chemically computed gas-phase free energies in this work refer to an ideal gas at 1 bar (denoted by °), free energies of solvation are defined via the hypothetical process of transferring 1 M of the solute from an ideal gas into an ideal solution at the same concentration (denoted by *). This change is included via the correction term

2 $$\Delta G^{{}^{\circ}\to *}=RT\ln\left(\frac{V_{0}}{V^{*}}\right)=RT\ln\left(24.79\right)=1.89kcal/mol$$

with the gas constant *R*, temperature *T* and the molar volume *V*_0_ of an ideal gas at 1 bar. A second correction term, a translational entropy correction, arises from the bottom reaction in Figure 2. The formation of a solute–solvent cluster from a solvated ion and a solvated solvent cluster is not associated with a change in Gibbs free energy. However, due to the definition of Gibbs free energies of solvation with a standard state of 1 M, the change of the Gibbs free energy of 1 mol of (S)_*n*_ gas from $\frac{\left[S\right]}{n}$ M to 1 M has to be considered (with [S] being the concentration of the pure solvent).

3 $$\Delta G_{{\left(S\right)}_{n}}^{l\to *}=-RT\ln\left(\frac{\left[S\right]}{n}\right)$$

More detailed explanations regarding the standard state, including interpretations in terms of translational entropies, can be found in ref 25.

**Figure 2:**
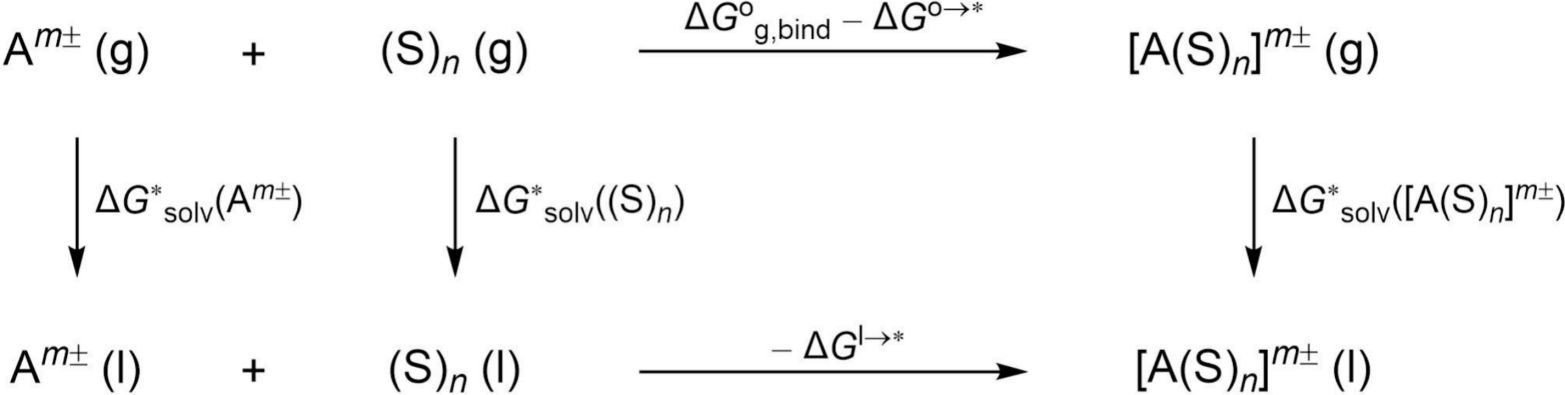
Thermodynamic cluster cycle for the calculation of $\Delta G_{solv}^{*}\left(A^{m\pm }\right)$ .

The cluster cycle has the advantage over a “monomer cycle” that it does more naturally account for the translational entropy of the solvent, it leads to a better cancellation of continuum-model errors, and it has been found to provide more realistic results in refs 25 and 39 . Indeed, the monomer cycle does not converge properly with increasing cluster size.^15,40,41^ It has also been stated that the use of single solvent molecules may generate artificial extra-thermodynamic assumptions.^42^ Many cluster-continuum calculations in the literature prefer the monomer cycle, as it is easier to compute just a single solvent molecule rather than neutral clusters of increasing size *n*. We will mention these works where appropriate (see below) but will not attempt to compare to their numerical values, due to these disadvantages and the resulting less accurate results.

For applying eq 1, $\Delta G_{g,bind}^{{}^{\circ}}$ can be calculated by a sufficiently accurate electronic-structure method together with thermodynamic contributions from statistical thermodynamics (typically using the harmonic-oscillator/rigid-rotor approach). $\Delta G_{solv}^{*}\left({\left[{A\left(S\right)}_{n}\right]}^{m\pm }\right)$ and $\Delta G_{solv}^{*}\left({\left(S\right)}_{n}\right)$ can be obtained from an implicit solvation model. If the implicit solvation model is capable of calculating $\Delta G_{solv}^{*}\left({\left[{A\left(S\right)}_{n}\right]}^{m\pm }\right)$ and $\Delta G_{solv}^{*}\left({\left(S\right)}_{n}\right)$ exactly, and our electronic-structure method gives the exact cluster binding energies, eq 1 gives the exact result independent of cluster size *n*. For infinitely large *n*, $\Delta G_{solv}^{*}\left({\left[{A\left(S\right)}_{n}\right]}^{m\pm }\right)$ and $\Delta G_{solv}^{*}\left({\left(S\right)}_{n}\right)$ become equal, and eq 1 reduces to

4 $$\Delta G_{g,solv}^{*}\left(A^{m\pm }\right)=\Delta G_{g,bind}^{{}^{\circ}}-\Delta G^{{}^{\circ}\to *}-\Delta G_{{\left(S\right)}_{n}}^{l\to *}$$

This equation can be understood as the calculation of the Gibbs free energy of solvation entirely from a microsolvated cluster model (indicated by the subscript g) without embedding into a continuum solvation model. Consequently, $\Delta G_{g,solv}^{*}\left(A^{m\pm }\right)$ should converge to the exact result for large *n* (as long as $\Delta G_{g,bind}^{{}^{\circ}}$ is exact). As the cluster-continuum approach is considered whenever one assumes that the quality of the implicit solvation model for the direct calculation of $\Delta G_{solv}^{*}\left(A^{m\pm }\right)$ is insufficiently accurate, also the quality of $\Delta G_{solv}^{*}\left({\left[{A\left(S\right)}_{n}\right]}^{m\pm }\right)$ and $\Delta G_{solv}^{*}\left({\left(S\right)}_{n}\right)$ will depend on the implicit embedding model. Therefore, the results obtained by eq 1 with different *n* will, of course, also depend on the model used.

Significant work using cluster-continuum models has been done for water, including limited studies on convergence of eqs 1 and 4 with cluster size *n*,^20,25,43,44^ often with relatively small clusters.^21,45^ Less work on convergence with cluster size has been done for other solvents.^15,46,47^ The goal of the present work is to study both the cluster-size dependence more systematically for a variety of anions and cations, to much larger cluster sizes than done hitherto, and in particular to include a variety of solvents. While construction of the clusters in most studies was usually done manually, albeit some Monte Carlo or MD simulations have been used as well,^15,43,46,48^ the use of a partially automated protocol for cluster generation and conformational selection (see Computational Details) should also lead to a more consistent progression of the computed free energies with increasing cluster size.

### 2.2 Single-Ion Scales for Gibbs Free Energies of Solvation

The introduced thermodynamic cycle allows the computation of Gibbs free energies of solvation for single ions. However, it is well-known that these are not fully defined experimentally. This is due to the unknown Galvani potential of transferring an ion from the gas phase to a solution phase. The Gibbs free energies of solvation for ion pairs, like those of neutral molecules, are independent of this contribution and straightforwardly measurable, as the potential cancels out between cation and anion. Ambiguities and “extra-thermodynamic assumptions” come into play when we want to compare to Gibbs free energies of solvation of individual ions. At least five types of single-ion solvation scales are discussed in the literature (conventional, bulk, intrinsic, real, and “absolute”). Notably, once one single-ion value can be fixed, the scale is defined and the values for other ions in the same solvent can be extracted from well-defined experimental free-energy differences.

We will not cover the discussion of the single-ion scales in all detail and refer the reader to refs 42 and 49–51 and references therein. The “conventional” scale sets the proton value to zero and then compares values for other ions to this. We will not consider this scale further. As the “bulk” scale is thought to exclude both the surface potential term and a local potential caused by creating a cavity for the ion in the solution, it appears more relevant for computations using classical force fields. Our cluster-continuum calculations clearly account for the local cavity potential, as the local environment of the ion is taken into account at the quantum-chemical level. A solvent scale that includes the surface potential in some way has been called “real” while a scale that does not include the surface potential but a local potential has been termed “intrinsic”.^49–51^ In some cases, scales have been labeled “absolute”, but this appears to mainly serve as a distinction from the conventional scale, and both real and intrinsic scales have been named “absolute”.^52,53^ We will therefore not use the “absolute” label but will mainly refer to “real” or “intrinsic” scales.

The most widely adopted single-ion solvation scale is that of Tissandier et al.,^54^ and of Kelly et al.,^55^ based on the cluster pair approximation (CPA). Combining experimental and computed binding energies for clusters of various ions with a small number *n* of explicit solvent molecules with further experimental data, these CPA scales determine the Gibbs solvation energy of the proton in water to −265.9 kcal/mol and thereby fix the zero of this particular scale (see Section 4.1.1 for CPA proton anchor values of other solvents). This CPA scale has been used, e.g., to parametrize the widely used SMD continuum solvent model.^13^ While the CPA has been designed explicitly to provide an intrinsic scale that excludes the surface potential,^42,54,55^ other works suggested that such a term is in fact included.^23,47^ While a pure cluster model does indeed exhibit an interface to the gas phase and may therefore include some surface contribution,^52,53,56^ a counterargument is that the relevant cluster sizes used are far too small to amount to a true surface potential.^42,57,58^ This would suggest the CPA to be close to an intrinsic scale. Notably, however, any intrinsic scale involves extra-thermodynamic assumptions, and only “real” single-ion solvation parameters are in principle measurable, albeit often with great experimental difficulty.^42^ Some of the approximations underlying the CPA have been put in doubt,^52,53,59^ also pointing to associated extra-thermodynamic assumptions.

The other widely used single-ion scale is the TATB scale.^60–63^ Its zero-point is fixed by assuming that the Gibbs energies of solvation of a large, roughly spherical cation (tetraphenylarsonium, TA^+^) and of an equally large anion (tetraphenylborate, TB^–^) are equal. This has been justified by the similar, large sizes of these ions and may be labeled a Born extra-thermodynamic assumption,^64^ as it asumes the purely electrostatic interaction between a rigidly spherical hard ion and a dielectric continuum. Then the ion-pair value for TATB is split in an exactly 50:50 way. The obtained values fix a scale for both cations and anions which has been thought to exclude the surface potential and to therefore also correspond to an intrinsic scale.^62^ We have applied the current cluster-continuum treatment also to TA^+^ and TB^–^ in water and in dichloromethane (DCM) and will examine the validity of the TATB assumption in Section 4.1.3 below.

Accurate cluster-continuum results based on the thermodynamic cluster cycle of Figure 2 constitute their own single-ion scales. Here the chosen cycle defines reference points for the potentials of the gas and liquid phases, which may also be viewed as setting a scale based on an extra-thermodynamic assumption.^42^ The question then arises, if the surface term is included in the cluster-continuum calculations, i.e., whether the resulting scale is real or intrinsic. Following the above discussion for the CPA, one may argue that the employed cluster sizes are too small to generate a macroscopic surface potential. Note that in contrast to the CPA the cluster-continuum model does not use an extrapolation to the bulk but rather combines a finite cluster of variable size and a continuum embedding model. Continuum solvent models like COSMO cover infinite space and do not exhibit a surface term.^25^ It has been argued that this extends also to the use of such continuum models for embedding the ion–solvent clusters.^27^ COSMO-RS also does not exhibit a term linear in the charge of a single ion like a surface potential. The Born-like polarization term of a dielectric continuum depends on the square of the ionic charge, which does not shift the single-ion scale. This holds also for the SMD model.^13^ But here we have a small caveat in that the “non-bulk-polarization” contribution of SMD, i.e., the “cavity-dispersion-solvent” structure term (*G*_CDS_),^13^ has been fitted also with the help of single-ion free energies of solvation, notably those based on the CPA scale. While there is no explicit term linear in ionic charge in the model, the surface tension terms in the CDS contribution might include an extra-thermodynamic assumption due to this fitting to single-ion data.^65^ We will examine the differences between the various embedding models in Section 4.

## 3 Computational Details

Figure 3 shows schematically the chosen semiautomatic workflow of our cluster-continuum modeling of Gibbs free energies of solvation, indicating also some variations made. Cluster structures were created with the QCG algorithm^28^ as implemented in CREST 2.12.^31,32^ The ensemble generation mode was used, whereas the number of solvent molecules was predefined stepwise. The NCI-MTD (noncovalent interactions-metadynamics) runtype used by default performed additional molecular dynamics (MD) runs at 1500 K for 50 ps to enhance the probability of getting a very diverse initial ensemble for subsequent DFT reoptimization. Unless noted otherwise, the GFN2-xTB tight-binding method^30^ was used for every step. In cases where this led to poor ensembles, we used the GFN-FF force field instead (see below). The temporary directories were saved. For more control over the sorting and exclusion of conformers, the temporary file before the last reduction step of QCG was used. The conformational ensemble contained therein was then sorted with the CREGEN algorithm of CREST, applying an energy threshold of 10 kcal/mol. We note that this part of the workflow is a computationally expedient replacement for much more expensive ab initio molecular dynamics suggested previously to select the most favorable conformers for cluster-continuum models.^66^

**Figure 3:**
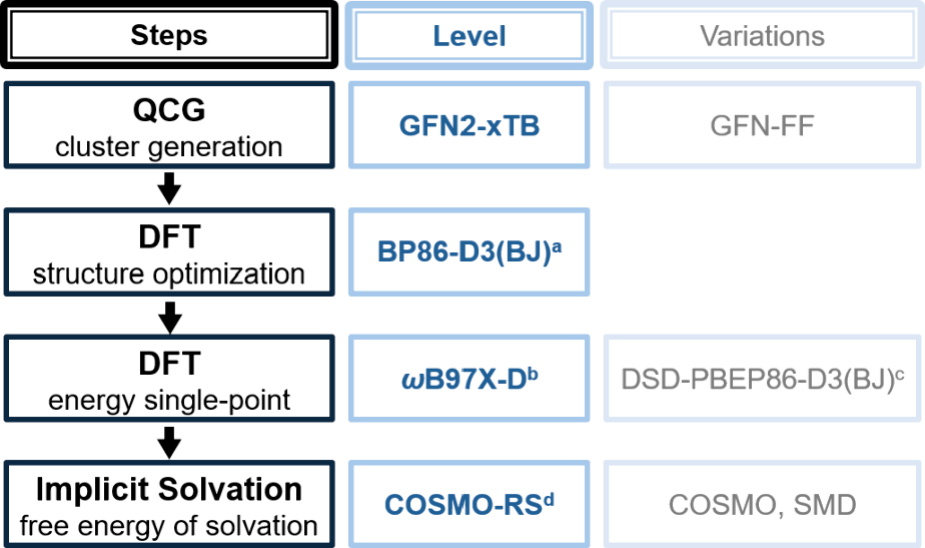
General workflow for the cluster-continuum model calculations of Gibbs free energies of solvation with some variations evaluated. Further possible variations are discussed in the text. ^*a*^Typically with def2-TZVP basis sets. This level is used also to compute internal thermodynamic contributions within the rigid-rotor/harmonic oscillator framework. ^*b*^Typically with def2-TZVPD basis sets. ^*c*^Typically with def2-TZVPD basis sets, but larger core–valence basis sets help improve results for alkali-metal cations like K^+^ (see text). ^*d*^We used by default the 2024 COSMO-RS parametrization of the COSMOtherm program but also evaluated other parametrizations (see text).

All DFT calculations were performed with Turbomole 7.5.1 ^67^ and a local version based on release 7.9.^68–70^ Up to 30 of the lowest-lying conformers were selected and reoptimized at the computationally expedient BP86 ^71–73^ -D3­(BJ)^74,75^ /def2-TZVP^76^ level. This number of conformers was largely dictated by the computational demands of the subsequent DFT calculations. While more conformers could in principle be used, the smooth size-dependent free energy curves obtained (see the Results section) support the choice. Structure optimizations employed a convergence criterion of 10^–7^ Hartree for the energy and 10^–4^ for the Cartesian gradient norm. Harmonic vibrational frequencies were calculated at the same level to ensure that the structures converged into local minima. Thermodynamic contributions at 298.15 K and 1 bar were calculated with the rigid-rotor/harmonic-oscillator approximation. We have not embedded the clusters in an implicit solvent model, either when generating them with QCG or during optimization, as has been done in some other studies.^26^ We have found that this has only a minor impact on the results (see data for fluoride in water in Supporting Information, Figure S1), and it would unnecessarily increase the computational cost. We note in passing that the r^2^SCAN-3c composite level^77^ would be a similarly useful alternative for this part of the workflow.

Subsequent energy single-point calculations were performed at the ωB97X-D^78^/def2-TZVPD^79^ level. The calculations employed a standard integration grid (gridsize 3) and the multipole-accelerated RI-J approximation^80^ together with the corresponding auxiliary basis sets.^81^ The energy convergence criterion was set to 10^–9^ Hartree ($scfconv 9), the density convergence to 10^–7^ ($denconv 1d-7).

The additional Gibbs free energy of solvation contributions due to the embedding of a given cluster were added at the COSMO-RS level using the COSMOtherm program,^14,82–84^ version 2024, with the BP-TZVPD-fine parametrization (BP_TZVPD_FINE_24). As an input for the COSMO-RS computations, additional single-point calculations at the gas-phase optimized structures were performed at the BP86/def2-TZVPD level in the gas phase and with COSMO (with the keyword $cosmo_isorad). The final free energies of solvation without or with embedding contribution were calculated by applying eq 1. The solvent densities employed can be found in Supporting Information (Table S1). The Gibbs free energy of a given conformer was taken as the sum of the electronic energy, the internal thermodynamic contributions of the cluster, and the embedding COSMO-RS contribution. Unless noted otherwise, the conformer with the lowest total Gibbs free energy was used, as we found Boltzmann averaging over conformers to not decisively influence the results (see the Results section).

In some cases, variations were made to the workflow (Figure 3) to preserve high accuracy in cases where the standard setup might be less appropriate. For the construction of fluoride-dichloromethane clusters, the GFN-FF^29^ force field had to be applied instead of GFN2-xTB to ensure that the ensemble generation does not lead to a substitution of chloride by fluoride (this is a reaction that indeed occurs over time experimentally^85^). The same was done for the solvation of the proton by MeCN, for the solvation of fluoride by acetone and DMSO (due to poor starting structures for subsequent DFT optimizations), and for the solvation of ${{\mathrm{AsPh}}_{4}}^{+}$ and ${{\mathrm{BPh}}_{4}}^{-}$ by water and DCM. For the solvation of the latter ions in DCM, just the 10 lowest-lying conformers of the QCG-generated ensemble were used because of an expected favorable convergence behavior and to keep computational cost affordable. Energy single-points for these larger ions were obtained at the ωB97X-D/def2-TZVP level, after ascertaining that the slight reduction of basis-set size does not substantially influence the results.

The standard ωB97X-D/def2-TZVPD level was found to not describe core–valence correlation in the group 1 cations with sufficient accuracy (see the Results section). Therefore, additional single-point calculations were performed with the DSD-PBEP86-D3­(BJ) double-hybrid functional and def2-TZVPD basis sets using the ORCA program,^87–89^ release 6.0.0, with the convergence criterion VeryTightSCF, the large grid DEFGRID3 and the RIJCOSX^90^ approximation. Even higher accuracy for the group 1 ions could be achieved with specific core–valence basis sets (see below). Of course the present workflow could be modified further by more expensive post-Hartree–Fock single-point energy calculations, but then application to larger cluster sizes is more limited. We will show below that in cases, where a direct comparison with literature CCSD­(T) results for smaller clusters is possible, the agreement is clearly within the margins of errors of the other computational aspects, certainly when using the double hybrid.

We also varied the embedding implicit solvent model in some cases to evaluate its effect. This included the simpler COSMO model (see Table S1 for the applied dielectric constants)^6^ or the SMD model,^13^ using in the latter case the implementation in ORCA. In a few cases we have also compared to other parametrizations of COSMO-RS, i.e., an older one^82^ from the COSMOtherm code referred to as “2012” (BP_TZVPD_FINE_HB2012_C30_1201), and the parametrization given in the openCOSMO-RS code as implemented in ORCA (openCOSMO-RS 24a).^91^ For comparison with the QCG-based cluster generation, we also evaluated in a few cases the SOLVATOR module in the ORCA code, version 6.0.0,^87,89^ for the initial generation of the clusters (usually at the GFN2-xTB^30^ level). Details are provided in the Supporting Information. DFT reoptimization, energy computations, and solvent embedding were subsequently done identically to the QCG-based computations (see above).

The Gibbs free energy of the proton in the gas phase was taken as *G*(H^+^) = −6.3 kcal/mol.^92^ We have not accounted for effects of Fermi–Dirac quantum statistics of the free electron in the formation of the proton, which would increase the free energy of solvation of the proton by ca. 0.9 kcal/mol.^42,58^

## 4 Results

### 4.1 Aqueous Solution

#### 4.1.1 The Proton in Water, Relation to Single-Ion Scales

Given its central role for several of the single-ion scales and for many derived quantities, e.g., in the context of redox potentials, we start with the proton in water. Figure 4 shows the application of the cluster-continuum workflow to the Gibbs free energies of solvation computed with increasing cluster size (based on eq 1), as indicated by the *n* explicit water molecules included. We compare results for the free gas-phase clusters to different embeddings. In contrast to the curve for the gas-phase clusters, the embedded-cluster curves start at much more realistic values already at *n* = 1, showing that the implicit solvent models cover substantial contributions. The gas-phase cluster results are not fully converged even at the largest *n* = 20. Indeed, the curve does not appear to converge to the asymptote of the embedded-cluster curves. We find such behavior also for other ions where the gas-phase cluster curve is already more flat. This shows clearly that the embedding contribution is appreciable and not easily covered by increasing *n* for the gas-phase clusters. Similar observations have been made before,^25,40^ and they pertain to both cations and anions. We note in passing, that in contrast to other ions studied in this work, for *n* ≥ 7 the proton tends to be localized on the exterior of a given cluster, even with larger *n*. This has been found before.^25,93,94^ Yet the embedded clusters provide only very modest oscillations in free energy beyond *n* = 3. In part this is due to the selection of the conformer with the lowest Gibbs free energy for each *n* (see Computational Details), which helps to smoothen the curves (this is shown explicitly below for other ions). We find that the ωB97X-D range-separated hybrid functional and the DSD-PBEP86 double-hybrid functional provide parallel curves differing only by a small systematic shift of 2.2 kcal/mol, with the double hybrid giving slightly less negative values. The different embedding models also affect the free-energy curves only moderately, with SMD giving somewhat more negative free energies.

**Figure 4:**
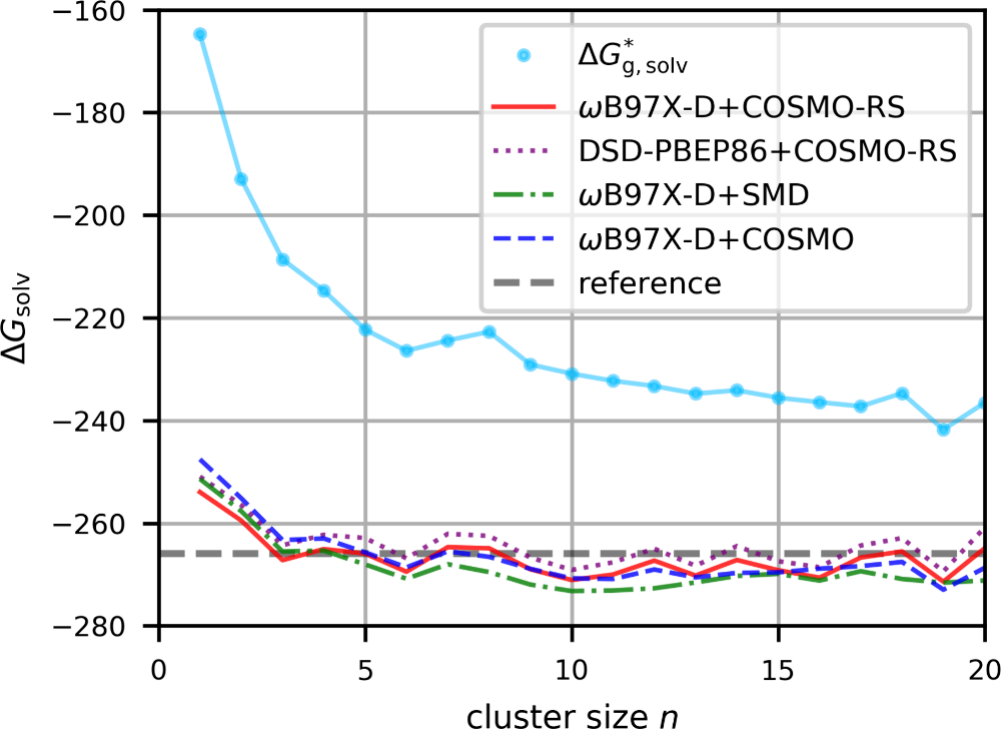
Gibbs free energy of solvation in kcal/mol of H^+^ in water calculated with gas-phase clusters $\left(\Delta G_{g,solv}^{*}\right)$ or with the cluster-continuum approach for different cluster sizes and different embeddings (SMD vs COSMO-RS) and different computational levels for the electronic energies (ωB97X-D range-separated hybrid vs DSD-PBEP86 double-hybrid functional). The conformer with the lowest total Gibbs free energy *G* ^tot^ has been used for each cluster size *n*. The reference value is the CPA value from ref 55. Circles for individual data points are shown for the gas-phase results but are omitted for clarity from the cluster-continuum curves.

Table 1 compares the averages over *n* = 9–17 to CPA- and TATB-based values, and also averaged values for the proton in other solvents, to be discussed later. The COSMO-RS-embedded values are close to the CPA-based proton value in water. The DSD-PBEP86 value is ca. 2 kcal/mol less negative than the ωB97X-D result and clearly within the stated CPA error margins of around 2–3 kcal/mol,^55^ while the cluster-continuum results differ much more from the TATB-based value. The SMD-embedded average value is 2.5 kcal/mol more negative than the COSMO-RS embedded one. Combination of SMD with the 2.2 kcal/mol less negative DSD-PBEP86 double-hybrid electronic energies brings us relatively close to the error margins of the CPA value. We will show below by comparison with ion-pair values that the present cluster-continuum computations may be considered to include uncertainties up to 3–4 kcal/mol, in most cases studied here only around 2 kcal/mol. The computed proton value in water is also close to that computed earlier with a cluster-continuum model without conformer selection, with *n* = 5, 9.^20^ Taking their best estimate at the CCSD­(T) level for *n* = 5 corrected to the present standard states,^25^ we arrive at −265.6 kcal/mol, which is clearly also within the error margins of the CPA-based estimates. An even earlier CCSD­(T)-based cluster-continuum value at *n* = 6 (when adjusted from 1 atm to 1 M in the gas phase) is −264.1 kcal/mol,^18^ and another cluster-continuum value of −266.7 kcal/mol^25^ using B3LYP electronic energies also fits within this framework when considering possible error sources. All three of these works used the cluster cycle we also adhered to here.

**Table 1:** Gibbs Free Energy of Solvation $\overset{®}{\Delta G_{solv}^{*}}$ in kcal/mol for the Proton in Different Solvents from the Cluster-Continuum Approach at Different Electronic-Structure and Embedding Levels in Comparison to CPA-Based and TATB-Based Reference Data

	range of *n*	ωB97X-D +COSMO-RS	$\Delta_{n}$ ^a^	DSD-PBEP86 +COSMO-RS	ωB97X-D +SMD	CPA	TATB
water	9–17	–269.0	2.3	–266.8	–271.5	–265.9^b^	–254.9^d^
MeCN	7–15	–251.9	1.8	–248.9	–257.7	–260.2^c^	–244.2^e^
MeOH	5–13	–260.0	3.0	–259.0	–270.2	–263.5^c^	–252.8^e^
DMSO	3–7	–272.4	0.8	–270.3	–280.4	–273.3^c^	–259.5^e^

a Maximum deviation from the average within the range of *n* (in kcal/mol) at the ωB97X-D level with COSMO-RS embedding.

b Reference 55 obtained −266.1 kcal/mol, confirming the earlier CPA value −265.9 kcal/mol of ref 54.

c Reference 95.

d Reference 62.

e From the water value of ref 62 and the solvent transfer values of ref 63.

Table 1 also provides proton values computed in the same way in MeCN, MeOH, and DMSO. The somewhat larger deviations for MeCN from the CPA-based scale of ref 95 will be discussed in Section 4.3 below. The plots for the dependence on cluster size and on electronic-structure level and embedding for the proton in methanol and DMSO are in Figures S2 and S3 in the Supporting Information, those for acetonitrile are in Figure 7. For MeOH and DMSO, agreement with the CPA-based values is similarly good as for water. SMD-embedding provides notably more negative values than COSMO-RS embedding, with the latter giving data closer to the CPA-based reference values. For methanol, the COSMO-RS-based values are about 3–4 kcal/mol less negative than the CPA values. The DSD-PBEP86 double hybrid gives about 1–3 kcal/mol less negative values than ωB97X-D also in these cases. In the methanol case we see a slight increase (Figure S2) of the COSMO-RS-embedded curves between *n* = 5–10, which is not visible with COSMO or SMD embedding. The trend likely reflects hydrogen bonding in the second solvation shell being present in COSMO-RS. Curves for the proton in DMSO are smooth and well-converged and also show the more negative values for SMD-embedding than for COSMO-RS-embedding (Figure S3). We suspect that this behavior of SMD is related to the use of single-ion data in its parametrization (see Section 2.2), which appears to introduce a shift to more negative values for cations and less negative ones for anions.

#### 4.1.2 Other Ions in Aqueous Solution

Due to its previously observed, strong charge-assisted hydrogen bonding interactions even in formally aprotic solvents,^15^ the fluoride ion is studied in particular detail here regarding the various computational aspects. Figure 5 shows the cluster-continuum solvation free-energy curves based on eq 1 for the gas-phase and COSMO-RS-embedded clusters of fluoride in water, up to *n* = 50 (Figure S4 in Supporting Information provides curves with different embedding solvent models). As for other ions, we see that COSMO-RS embedding leads to an excellent starting point already at *n* = 1, while without embedding (blue curve) a steep slope is seen at the beginning. The values for *n* = 1 with different embeddings (Figure S4) reflect the performances of the pure implicit solvent models (i.e., for *n* = 0; see Table S2 in the Supporting Information). In this case, the two COSMO-RS variants give somewhat more negative results than the converged cluster-continuum results, while COSMO and SMD start at less negative free energies of solvation.

**Figure 5:**
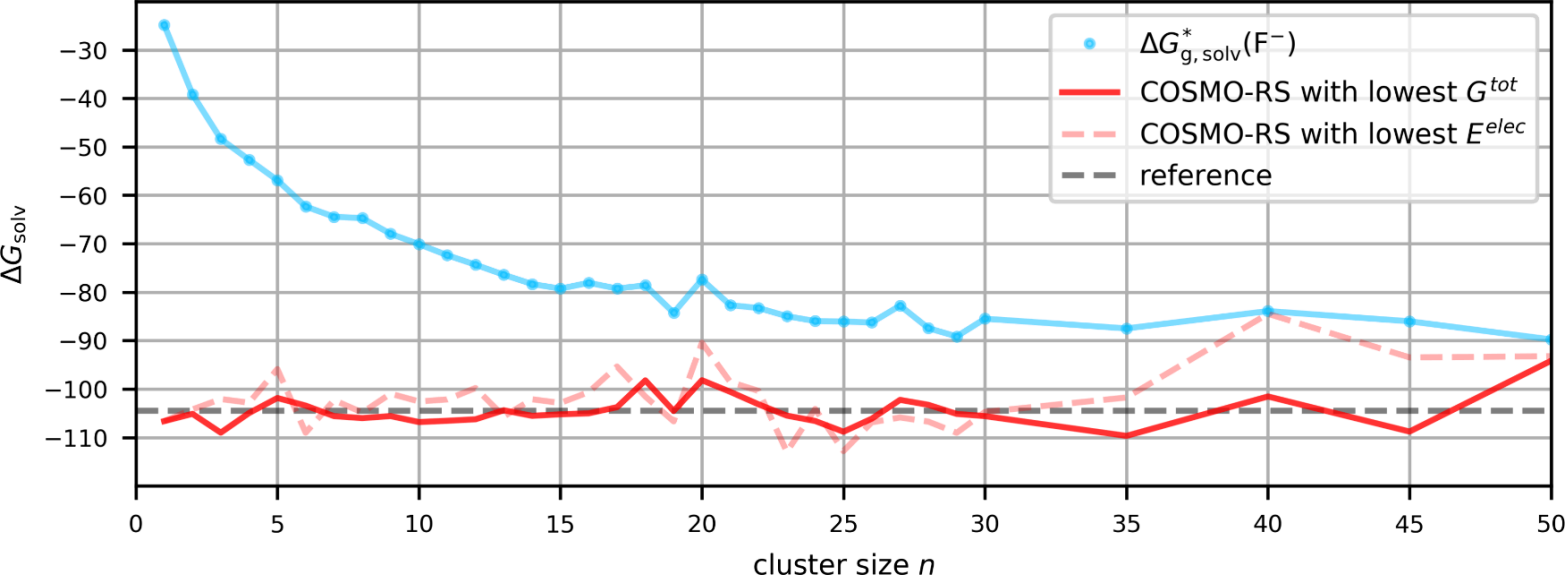
Gibbs free energy of solvation in kcal/mol of F^–^ in water calculated with the cluster-continuum approach for different cluster sizes. Results are shown without contributions from the implicit solvation model COSMO-RS $\left(\Delta G_{g,solv}^{*}\left(F^{-}\right)\right)$ and with the COSMO-RS contribution $\left(\Delta G_{solv}^{*}\left(F^{-}\right)\right)$ using the conformer with the lowest electronic energy *E* ^elec^ or with the lowest total Gibbs free energy *G* ^tot^. The CPA-based reference value is taken from ref 55.

It is seen generally that a selection of conformers according to the lowest overall free energy (solid red curve in Figure 5) leads to much smaller oscillations with *n* than when the choice is made with respect to the lowest electronic energy (dashed red curve). Obviously, contributions beyond the electronic energy can be decisive in determining the lowest-lying conformer. Selection based on lowest electronic energy is thus not recommended, although selection according to free energies requires us of course to compute all contributions for all low-lying conformers and is thus more computationally demanding. When using this more stable procedure, we see a plateau with almost constant free energy between *n* = 7–16. Increased scattering is seen for larger clusters. This arises from the enormously growing number of degrees of freedom for large clusters, which makes it very hard to find the global minimum structure or even an ensemble of very low-lying structures. We also note the first visible kink in the gas-phase cluster curve at *n* = 8. The reason is that 8 water molecules can form a stable neutral cube-like structure in the gas phase which is not possible for the fluoride-water cluster at *n* = 8 due to the additional vertex. Thus, the two structures are not well comparable, resulting in an underestimation of the binding energy $\Delta G_{g,bind}^{{}^{\circ}}$ . The cluster-continuum curve based on the conformer with lowest free energy does not exhibit the kink. It furthermore seems that the COSMO-RS embedding smoothens the differences.

Some scattering in the range *n* = 1–5 reflects the buildup of the first solvation shell (which is known to contain 4–6 water molecules^15,44,96–98^ ). This illustrates the two competing requirements on cluster size: a) the strongest solute–solvent interactions should be covered within the cluster; b) too large cluster sizes lead to a large computational burden and a complex conformational landscape, which we strive to avoid. This guides us naturally to a region with intermediate *n*, for the fluoride-water case to *n* = 7–16, where no individual value deviates by more than 1.3 kcal/mol from the average. We may in principle select such a range by eye or formalize selection by weighing the aim of small deviations over the given range against our wish to have a large size of the range. We chose a weighing function that is given in eqs S2 and S3 in the Supporting Information but note that the final average solvation free energies obtained from a visual selection are closely similar. The resulting range and average for fluoride in water are included in Table 2. Similarly to the proton value, the average agrees excellently with the CPA-based value from ref 55, less well with the TATB-based scale of ref 62. As for the proton (see above), the gas-phase cluster binding-energy curve appears to converge against a less negative value, indicating that even up to these cluster sizes, the gas-phase clusters do not recover the long-range charge polarization contributions. Table 2 includes also the “pure” COSMO-RS value of the isolated ion. The fact that it is also very close to the converged cluster-continuum value shows that for fluoride in water the chosen COSMO-RS parametrization is excellent, in particular regarding the description of the strong hydrogen bonds to fluoride. Comparison with an older 2012 parametrization of COSMO-RS (Table S3 in Supporting Information) shows that the value for fluoride has been improved by reparameterization. That is, for particularly strong hydrogen bonds like those to fluoride the parametrization of the model may be crucial,^84^ and we find significant changes also for, e.g., Li^+^. As we increase *n* for the cluster-continuum models, this parameter-dependence becomes much lower, however.

**Table 2:** Gibbs Free Energy of Solvation $\overset{®}{\Delta G_{solv}^{*}}$ in kcal/mol for Different Simple Ions in Water from the Cluster-Continuum Approach (at the *ω*B97X-D/def2-TZVPD + COSMO-RS Level) in Comparison to Pure COSMO-RS Calculations and CPA-Based and TATB-Based Reference Data

solute	$\overset{®}{\Delta G_{solv}^{*}}$ ^a^	Δ_max_ ^b^	range of *n*	Δ_*n*_ ^c^	$\Delta G_{solv}^{COSMO‐RS}$ ^d^	CPA^e^	TATB^f^
F^–^	–105.7 (−103.8)^g^	6.2	7–16	1.3	–106.1	–104.4	–114.9
Cl^–^	–74.3	10.7	10–15	0.5	–75.6	–74.5	–85.0
Br^–^	–67.9	7.8	9–17	1.9	–75.8	–68.3	–79.1
I^–^	–59.0	8.5	9–18	2.9	–70.2	–59.9	–69.5
H^+^	–269.0 (−266.8)^g^	11.9	9–17	2.3	-	–265.9	–254.7
Li^+^	–125.1 (−129.4)^g^	24.5	12–19	3.2	–97.3	–128.4	–117.3
Na^+^	–100.4 (−106.7)^g^	14.3	10–17	1.5	–87.8	–103.2	–91.0
K^+^	–79.8 (−83.9)^g^	15.4	8–16	2.3	–71.5	–86.0	–74.3
${{\mathrm{NH}}_{4}}^{+}$	–83.2	8.5	6–10	0.4	–82.7	–85.2	–71.9

a Average over a range of cluster sizes *n* with small oscillations.

b Maximum deviation in the range *n* = 1–20 from the CPA-based reference value (in kcal/mol).

c Maximum deviation from the average within the range of *n* (in kcal/mol).

d “Pure” COSMO-RS result. See Table S2 in the Supporting Information for a number of corresponding “pure” COSMO and SMD values.

e Reference 55.

f Reference 62. We had to apply a correction of −3.78 kcal/mol to these data, as it appears that the correction from 1 bar to 1 M in the gas phase (eq 2) had been used with the wrong sign.

g Results in parentheses used the DSD-PBEP86 double hybrid for the electronic energies instead of ωB97X-D.

As described in the Computational Details, for each *n* we used the cluster conformer with the lowest total Gibbs free energy. The QCG algorithm produces not just one microsolvated structure, but an ensemble of structures. Since the solution phase is highly dynamic, it is of course desirable to include as many and as diverse structures as possible. We tested the influence of including an ensemble of the 30 lowest conformers (cf. Computational Details) into the calculation of the Gibbs free energy of fluoride in water. This affects results for individual cluster sizes by less than 0.5 kcal/mol (see Figure S5 in the Supporting Information) which is clearly below the variation with cluster size. We find such behavior also with other ions. This shows that it is most important to find a conformer near the global minimum, which then is better than using an ensemble of unknown quality.

Analogous cluster-continuum curves with COSMO-RS embedding, and in some cases with alternative embeddings, for other simple ions are shown in Figure S6 in the Supporting Information. As oscillations increase for larger clusters (see above), we concentrate on the range *n* = 1–20. The free energies of solvation averaged over the obtained cluster sizes, $\left(\overset{®}{\Delta G_{solv}^{*}}\right)$ , are included in Table 2. The picture for the other halides, Cl^–^, Br^–^ and I^–^ is similar to that for fluoride, i.e., the oscillations with *n* over the optimal range are small, and the final average is in excellent agreement with the CPA-based scale, again within the error margins of the latter. Pure COSMO-RS without explicit solvation performs well for Cl^–^ but tends to be increasingly too negative for the heavier halides.

The pure COSMO-RS values are above the CPA values for the alkali-metal cations by an even larger amount. Here a description of the direct coordination of water oxygen atoms to the cation in the first solvation shell obviously is crucial but unlikely to be fully covered in the implicit model. A part of this behavior has been previously suggested to be linked to inaccuracies in the underlying proton value implied by COSMO-RS,^99^ but this assumption does not fit our overall observations for cations and anions (see below).

The cluster-continuum values for the group 1 cations are much closer to the CPA-based scale but tend to deviate somewhat more for the heavier ions, in particular for K^+^. We tested if this is due to an insufficient description of core–valence electron correlation^100–102^ for these very polarizable cations by the chosen ωB97X-D functional and repeated the calculations with the DSD-PBEP86 double hybrid. Its MP2 correlation part is expected^103–105^ to recover more of these contributions (see values in parentheses in Table 2). Indeed, this shifts the solvation free energies to more negative values for Li^+^, Na^+^, and K^+^, and brings them close to the CPA-based reference values. In the case of K^+^ the employed def2-TZVPD basis set is not fully converged regarding such core–valence correlation contributions when using the double hybrid. We therefore evaluated use of a larger aug-cc-pwCVQZ-X2C basis set^106^ for K^+^, specifically designed to recover core–valence contributions, for the electronic binding energy of a cluster with *n* = 5. Indeed, this increases the binding energy by another 2.5 kcal/mol. Such a shift brings the computed DSD-PBEP86 free solvation energy for K^+^ into perfect agreement with the CPA-based value. Table 2 also includes ${{\mathrm{NH}}_{4}}^{+}$ as an example for a polyatomic cation. The computed average deviates only by 2.0 kcal/mol from the CPA-based scale.

For some of these ions in water, we have also evaluated the SOLVATOR/GOAT scheme in the ORCA code (see Computational Details) for the initial conformer generation. Details are provided in the Supporting Information, see Figure S7 and Table S4. Here we only note that the overall results are similar as those obtained from the QCG-based sampling.

Table S5 in the Supporting Information provides also the computed solvation free energies of ion pairs in water, calculated from the combination of the single-ion values in Table 2 at the ωB97X-D/def2-TZVPD level with COSMO-RS embedding. Here the comparison with experiment is straightforward without any extra-thermodynamic assumptions. Agreement with experiment is typically 2–4 kcal/mol, except for the potassium salts where the chosen electronic-structure level gives systematically too positive ion-pair values by about 5–7 kcal/mol. Using the more negative values for the alkali metal cations at the DSD-PBEP86 double-hybrid level (Table 2) improves the results for the group 1 salts further. In particular, after correcting for core–valence correlation contributions of about −6.5 kcal/mol for K^+^ (see above) the values for potassium salts exhibit very small deviations from experiment as well. In contrast, if we add up the best single-ion cluster values without embedding into a solvation model, we get ion pair values that are too positive by ca. 45–69 kcal/mol (Table S5). This shows clearly that the long-range polarization contributions supplied by the embedding are crucial.

A number of other cluster-continuum or QM/MM calculations are available in the literature for some of the ions covered here in aqueous solution, usually just for one or a few ions, and with widely varying computational methods.^20,23,40,46,47,107^ As most of these studies used the less suitable monomer cycle (see the Theoretical Background section) and relatively small *n*, we will refrain from a direct comparison to all of these data. Note, however, that the value for Li^+^ obtained with *n* = 4 at the ab initio composite level (CCSD­(T) with an MP2 correction to extrapolate to the complete basis set) within a cluster cycle in ref 20 is −128.5 kcal/mol when corrected to 1 M standard states, in excellent agreement with our best computations at the DSD-PBEP86 level and with the CPA-based scale (Table 2). We also note extensive QM/MM MD simulations (with the first hydration shell described at the MP2 level) of the alkali-metal cations,^104,105^ which gave values for Li^+^ (−125.5 kcal/mol), Na^+^ (−99.7 kcal/mol), and K^+^ (−82.1 kcal/mol) and used those values to then extract a proton value of −262.9 kcal/mol. All of these data are somewhat less negative than the CPA-based values or the best present cluster-continuum values (Table 2).

#### 4.1.3 Evaluation of the TATB Assumption Using the Cluster-Continuum Approach

We have mentioned above the significant deviations between the CPA- and TATB-based scales in water. The underlying assumption of the TATB scale,^62,108^ i.e., the equality of the free energies of solvation of ${{\mathrm{AsPh}}_{4}}^{+}$ and ${{\mathrm{BPh}}_{4}}^{-}$ , has been discussed controversially in the past. Based on classical MD simulations in water, a much more negative Gibbs free energy of solvation for the anion by ca. 20 kcal/mol was suggested using the TIP3P water model.^109,110^ That value reduced to merely 4.3 kcal/mol with the more charge-symmetrical^53^ TIP5P model. Pollard and Beck suggested that the large difference was due to an inconsistency in the contributions from the surface potential.^49^ The discussion involves the question whether the TATB scale is an intrinsic one that excludes the surface potential. Calculations using continuum solvent models also suggested that ${{\mathrm{BPh}}_{4}}^{-}$ is more strongly solvated than ${{\mathrm{AsPh}}_{4}}^{+}$ or ${{\mathrm{PPh}}_{4}}^{+}$ , by about 6 kcal/mol.^111,112^ A more recent ab initio molecular dynamics simulation study pointed to larger vibrational shifts for the anion compared to the cation as an argument for the stronger solvation of the former,^64^ and a variety of spectroscopic and thermochemical studies also support a different solvation of the two ions (see, e.g., refs 113–116 ).

Here we apply straightforwardly the cluster-continuum model separately to ${{\mathrm{AsPh}}_{4}}^{+}$ and ${{\mathrm{BPh}}_{4}}^{-}$ in water and in DCM (DCM was chosen for comparison as an aprotic solvent, and because we expected smoother curves with growing *n*). The computed curves in water are shown in Figure 6. They are not as smooth as would be desirable. Obviously, at the chosen cluster sizes up to *n* = 20, appreciable variation still occurs, as we possibly just about transcend the first solvation shell for these large ions. Nevertheless, for all three embedding models it is apparent that ${{\mathrm{BPh}}_{4}}^{-}$ exhibits more negative values than ${{\mathrm{AsPh}}_{4}}^{+}$ at any value of *n*. However, the magnitude of the differences does depend on the embedding model. Table 3 provides the corresponding average values. The table also includes results for the same cluster sizes without embedding, providing already a ca. 9 kcal/mol more negative value for the anion than for the cation. COSMO-RS embedding gives a larger difference of almost 20 kcal/mol, COSMO about 11 kcal/mol, and SMD a smaller one of 7 kcal/mol. The latter seems consistent with our general observation that SMD produces more negative values for cations and less negative ones for anions (also in MeCN, see below). This may arise from extra-thermodynamic assumptions introduced into SMD by the parametrization of its G_CDS_ term against ionic solvation free energies (see Section 2.2). Notably, the footnote to Table 3 also shows the pure implicit solvation free energies of the two ions without an explicit inclusion of water molecules. Here SMD even would suggest ${{\mathrm{BPh}}_{4}}^{-}$ to be less well solvated than ${{\mathrm{AsPh}}_{4}}^{+}$ . Such a result contradicts all of the accumulated experimental and computational evidence discussed above. In contrast, the pure COSMO-RS values suggest a ca. 13 kcal/mol larger solvent stabilization of the anion. These differences between COSMO-RS and SMD are also apparent for the starting values of the free-energy curves at *n* = 1 (Figure 6).

**Figure 6:**
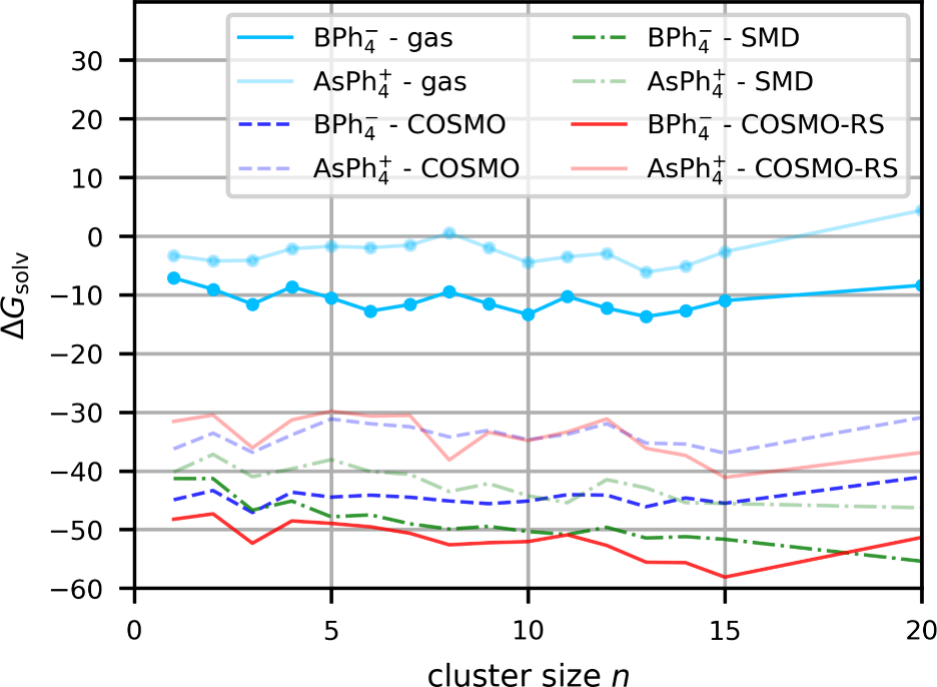
Gibbs free energy of solvation in kcal/mol of ${{\mathrm{AsPh}}_{4}}^{+}$ and ${{\mathrm{BPh}}_{4}}^{-}$ in water obtained with the cluster-continuum approach (ωB97X-D/def2-TZVP) for different cluster sizes and for different implicit embedding solvation models.

**Table 3:** Gibbs Free Energies of Solvation in kcal/mol for ${{\mathrm{AsPh}}_{4}}^{+}$ and ${{\mathrm{BPh}}_{4}}^{-}$ in Water and Dichloromethane Obtained from Cluster-Continuum Calculations (*ω*B97X-D/def2-TZVP) without Embedding and with Different Implicit Embedding Solvent Models^a^

solvent	ion	range of *n*	COSMO-RS	$\Delta_{n}$ ^b^	gas phase^c^	COSMO	SMD
water	${{\mathrm{AsPh}}_{4}}^{+}$	3–13	–33.0	3.2	–2.7	–33.6	–41.8
	${{\mathrm{BPh}}_{4}}^{-}$	7–12	–51.9	1.2	–11.4	–44.8	–49.9
DCM	${{\mathrm{AsPh}}_{4}}^{+}$	2–8	–40.3	1.3	–3.9		
	${{\mathrm{BPh}}_{4}}^{-}$	7–15	–62.9	2.9	–36.5		

a Calculations without explicit solvation in water at the ωB97X-D/def2-TZVPD level give −47.2 and −44.9 kcal/mol with SMD and −33.0 and −46.1 kcal/mol with COSMO-RS for ${{\mathrm{AsPh}}_{4}}^{+}$ and ${{\mathrm{BPh}}_{4}}^{-}$ , respectively.

b Maximum deviation from the average within the range of *n* (in kcal/mol) at the ωB97X-D level with COSMO-RS embedding.

c No embedding.

Overall COSMO-RS is preferable here due to its known realistic local description of hydrogen bonding contributions.^99,117,118^ Its lack of parametrization to ion data also seems an advantage in this context. When used within the cluster-continuum approach, we expect COSMO-RS embedding to be preferable over the COSMO or SMD embedding. We also note the excellent COSMO-RS embedded results for other ions in aqueous solution (see above), as well as the good performance for ion pairs. The larger difference between ${{\mathrm{AsPh}}_{4}}^{+}$ and ${{\mathrm{BPh}}_{4}}^{-}$ in water for COSMO-RS embedding therefore seems realistic. Note also, that the sum of the TA^+^ and TB^–^ values at this level is ca. −85 kcal/mol, which may be taken as a reasonable prediction for the TATB pair value. This exergonic value should be contrasted to an endergonic one of about +24 kcal/mol provided as two positive single-ion values of +12 kcal/mol in ref 62. Even the sum of the latter two values involves a number of assumptions with large uncertainties. For example, the enthalpy contributions involve lattice energies of the corresponding salts,^119^ which may not be reliable.^120^ Furthermore, transfer from the conventional to the TATB scale involves also proton entropy and enthalpy contributions with uncertainties.^62,121,122^

Assuming that our result of a ca. 20 kcal/mol more negative ${{\mathrm{BPh}}_{4}}^{-}$ Gibbs free energy of solvation compared to ${{\mathrm{AsPh}}_{4}}^{+}$ (Table 3) is correct, we have to shift all TATB values (from ref 62, corrected for an erroneous standard-state correction) in Table 2 above for cations to ca. 10 kcal/mol less negative and those for anions to 10 kcal/mol more negative values. This clearly moves this scale even further away from the CPA-based scale and from the present cluster-continuum results, as had been suggested earlier.^120^ That is, one arrives at a larger shift between the two scales than hitherto, ca. 20 kcal/mol with COSMO-RS embedding, ca. 16 kcal/mol with COSMO embedding. If we proceed under the premise that both the CPA-based and the TATB-based scales both correspond to intrinsic scales, and such intrinsic single-ion Gibbs free energies of solvation have no operational meaning,^42,52,53,123,124^ it is clear that their underlying extra-thermodynamic assumptions differ substantially. This holds with the original TATB scale but even more so if we accept the necessity of correcting it. We note in passing, that the TATB hypothesis is used in various fields, including the transfer of ions through membranes in biophysics.^111^

Maybe more importantly, the excellent agreement of the present cluster-continuum single-ion Gibbs free energies of solvation with the CPA-based scale suggests that the underlying extra-thermodynamic assumptions of these two scales are closely aligned, in spite of their different origins. The good agreement between cluster-continuum calculations and the CPA-based scale had been noted earlier.^52,53^ As mentioned further above, the question if a cluster model includes a surface potential has been discussed controversially.^42,52,53,56–58^ Assuming that the relevant cluster sizes of either CPA or the present cluster-continuum models are too small to generate a macroscopic surface potential,^42,57,58^ both scales may be characterized as intrinsic. The fact that they produce closely aligned scales would then support that their underlying extra-thermodynamic assumptions are very similar.^52,53^ Notably, while the CPA-based scale is anchored to the proton value (see above) and uses otherwise experimental differences between different ions,^54,55^ the present theoretical cluster-continuum values have been computed independently for each ion in Table 2, without resorting to experimental information. This lends a certain degree of robustness to the present scale and, by inference, to the CPA-based scale. We will provide values with comparable accuracy for other solvents further below.

Coming back to the obviously different solvation of ${{\mathrm{BPh}}_{4}}^{-}$ and ${{\mathrm{AsPh}}_{4}}^{+}$ in water, we have also examined the hydrogen bonding of water to the π-system of the phenyl rings in both cation and anion in our DFT-optimized cluster structures and can confirm the clearly closer interactions for the anion, in agreement with the findings in ref 64. Figure S8 in the Supporting Information shows for a chosen cluster size *n* = 20 up to 14 notable hydrogen bonds from water molecules toward the phenyl rings for the anion, but just one for the cation. For *n* = 15 the difference is somewhat less pronounced, with 10 hydrogen bonds for the anion compared to 4 for the cation. In any case, we can clearly see the origins of the more negative Gibbs free energy of solvation of the anion compared to the cation in terms more pronounced hydrogen bonding for the former, consistent with previous ab initio molecular dynamics results.^64^ That is, the Born assumption does not hold due to local interactions^125^ that are in part nonelectrostatic.

For DCM, where convergence is smoother and seems to be reached already at *n* = 7 (see Figure S9 in the Supporting Information), we find a larger difference of almost 30 kcal/mol from the gas-phase clusters, which is in this case *reduced* to 20 kcal/mol upon COSMO-RS-embedding (Table 3). These results suggest that the TATB-assumption is also violated for aprotic solvents. If the ca. 20 kcal/mol more negative free energy of solvation of ${{\mathrm{BPh}}_{4}}^{-}$ compared to ${{\mathrm{AsPh}}_{4}}^{+}$ holds also for other solvents, it may leave the widely used TATB-based solvent transfer free energies for single ions from water to other solvents (see, e.g., ref 63) unaffected, even if the TATB assumption itself should be corrected.

### 4.2 Gibbs Free Energies of Solvation in Acetonitrile

Given the small size of the water molecule, one might consider aqueous solution as particularly simple for a cluster-continuum approach. However, due to the rigid and extended hydrogen-bonding networks in water it may actually be more difficult to converge cluster size than with other solvents. We consider again first the proton due to its use as an anchor point of the CPA-based scale. Figure 7 shows that very flat cluster-continuum free-energy curves are obtained over a large range of cluster sizes. The variation with the choice of solvent model used for embedding, and with electronic-structure method is significant, again with SMD embedding giving more negative values for cations as found above in aqueous solution (see above). A comparison of averaged values is included in Table 1 above, together with proton values in other solvents. For MeCN, the COSMO-RS-based results are about 10 kcal/mol less negative than the suggested CPA data from ref 55, giving significantly larger deviations from that scale than for any of the other solvents considered here. An application of TATB-based proton transfer free energies^63^ from water to MeCN to the water CPA value^55^ would produce a less negative proton free energy of solvation (ca. −255 kcal/mol) than the CPA MeCN value in the table, in much better agreement with our best cluster-continuum values. If the necessary corrections to the TATB scale are indeed similar in different solvents (see above), the transfer values are expected to be reliable. It thus seems possible that the CPA-based proton Gibbs free energy of solvation in this particular solvent might have to be adjusted to a less negative value (see also below). Another cluster-continuum study^26^ came to similar values as those computed here. Their directly computed values for MeCN and DMSO are −254.3 and −269.6 kcal/mol, respectively, when adjusted to the present standard states, while their application of solvent-transfer data to the water CPA value led them to experimental values of −254.8 and −270.5 kcal/mol, respectively (adjusted), in good agreement with our best values in Table 1. Their observation of typically six solvent molecules in a strongly bound core cluster also agrees with our own observation of relatively smooth and flat free-energy curves after *n* = 6. We find that two MeCN molecules coordinate directly to the proton and the further solvent molecules then bind to these central two.

**Figure 7:**
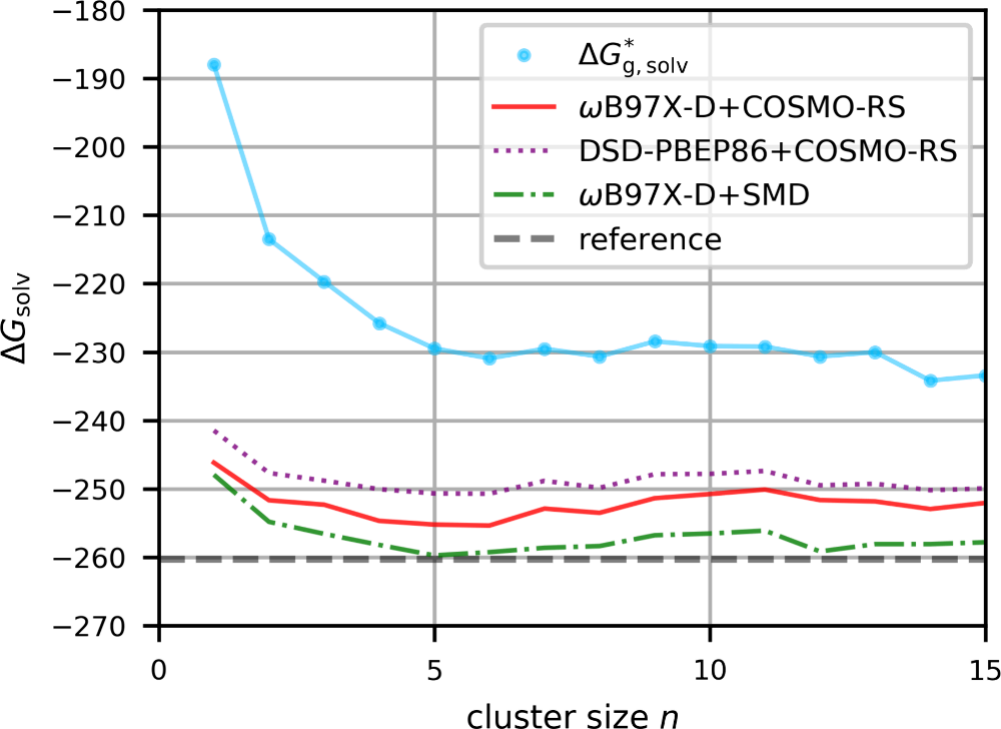
Gibbs free energy of solvation in kcal/mol for H^+^ in MeCN calculated with the cluster-continuum approach for different cluster sizes, and different embeddings and electronic energies.

Figure 8 shows the computed, rather smooth curves within the present QCG-based computational protocol for fluoride in MeCN, comparing different embeddings. Here SMD produces clearly less negative values than the other embedding models (as for other anions), again possibly caused by its intrinsic parametrization to ions (see above). Our previous work^15,126^ suggested the first solvation shell to be completed around *n* = 8, 9. For *n* values larger than this, we see flat and smooth curves. The resulting average (*n* = 7–16, Table 4) of −94.1 kcal/mol compares well with the best DLPNO-CCSD­(T)-F12 value for *n* = 7–10 of −93.1 kcal/mol from ref 15 (when corrected by eq 2). We note in passing that various DFT calculations in the same paper produced a ca. 10 kcal/mol more negative value. This is due to the lack of diffuse functions in the DFT basis set used, which is known to lead to errors in fluoride binding.^127^ No direct experimental value is available. Taking the CPA-based value for fluoride in water of −104.4 kcal/mol from Table 2 and an estimated TATB-based value for the free energy of transfer from water to MeCN of +10.8 kcal/mol,^128^ we arrive at −93.6 kcal/mol. This fits well with the present computations (Table 4) and may thus also support our proton value, which is less negative than the CPA-based proton value for MeCN^95^ (Tables 1 and 4). This shift from the CPA-based scale is apparent also for the other halide ions, where our best averaged cluster-continuum values (over smooth and flat curves, see Figure S10 in the Supporting Information) are about 8–10 kcal/mol more negative than the CPA-based ones (Table 4). For Li^+^ and Na^+^ the curves have a pronounced dip at *n* = 5 indicating the closure of the first solvation shell (Figure S10). If we use the values at this dip instead of a simple average over a larger range of *n*, we arrive at values of −124 and −102 kcal/mol, respectively, somewhat more negative than the average values listed in Table 4. It has been argued that one should choose the cluster size with the lowest free energy for cluster-continuum solvation models, as the continuum solvent model fails to capture the specific interactions for larger clusters, and therefore the free energy should rise again at larger *n*.^21^ We feel that with COSMO-RS embedding, this argument does not hold true, and we do see very flat curves over an appreciable range of *n* in many cases (see above). While no experimental value is available for Li^+^, our listed value for Na^+^ is only ca. 3 kcal/mol “too positive”, and the lower value at the dip would fit the suggested CPA reference value (Table 4), further complicating our comparison with that scale. We note also, however, the importance of core–valence correlation^100–102^ for the alkali-metal cations (see above for the aqueous case), a contribution that is not converged in the computed number (Table 4) for Na^+^. In any case, we suggest tentatively that the CPA-based proton value in MeCN might have to be corrected to be less negative by at least 5 kcal/mol.

**Figure 8:**
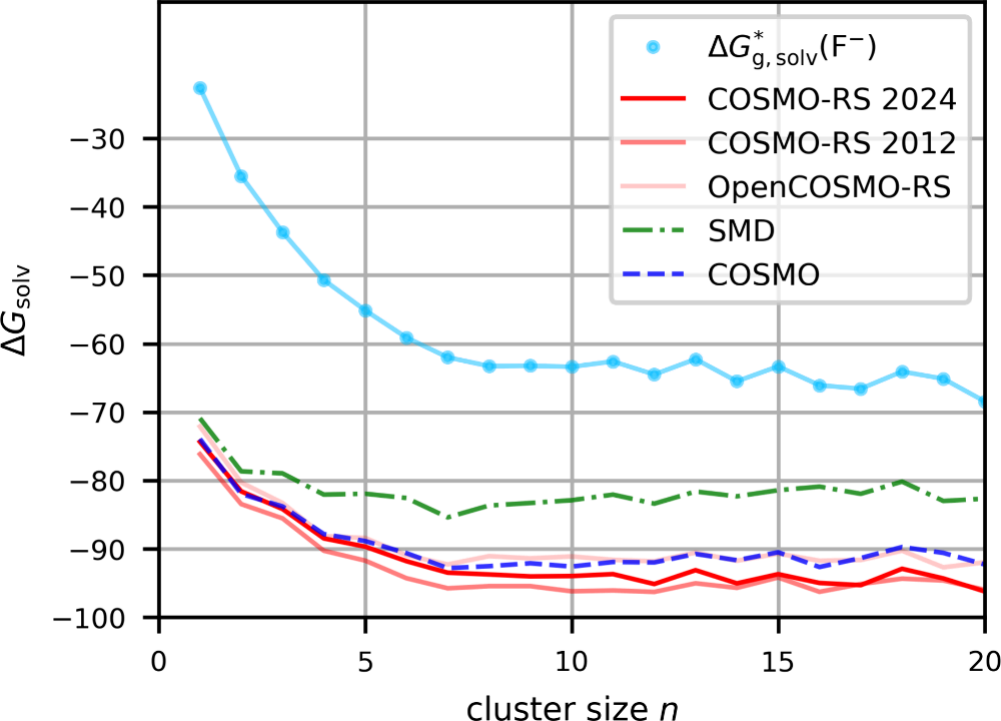
Gibbs free energy of solvation in kcal/mol for F^–^ in MeCN calculated with the cluster-continuum approach for different cluster sizes and different embeddings.

**Table 4:** Gibbs Free Energy of Solvation $\overset{®}{\Delta G_{solv}^{*}}$ in kcal/mol for Different Simple Ions in MeCN from the Cluster-Continuum Approach (at the *ω*B97X-D/def2-TZVPD + COSMO-RS Level) in Comparison to Pure COSMO-RS Calculations and CPA-Based Reference Data

solute	$$\overset{®}{\Delta G_{solv}^{*}}$$	range of *n*	$\Delta_{n}$ ^a^	$$\Delta G_{solv}^{COSMO‐RS}$$	CPA^b^
F^–^	–94.1	7–16	1.1	–76.2	-
Cl^–^	–72.8	9–17	1.6	–68.2	–62.4
Br^–^	–68.5	8–17	1.5	–66.3	–59.3
I^–^	–60.8	2–10	1.3	–63.5	–53.0
H^+^	–251.9	7–15	1.8	-	–260.2
Li^+^	–117.5	7–13	0.9	–95.4	-
Na^+^	–97.6	7–15	2.1	–86.9	–101.1

a Maximum deviation from the average within the range of *n* (in kcal/mol).

b Reference 95.

### 4.3 Solvation of Fluoride in Different Solvents

The strong charge-assisted hydrogen bonds to fluoride in MeCN^15^ beg the question about the occurrence of such hydrogen bonding in other aprotic solvents. The free-energy curves with increasing *n* for the additional solvents MeOH, acetone, DMSO, DCM, benzene, and diethyl ether are shown in Figure 9 (cf. Figures 5 and 8 for water and MeCN, respectively), the average values are compared in Table 5. No reliable reference values are available in these other solvents, but given the excellent agreement of our computations with the CPA scale in water (Table 2), the present cluster-continuum values may serve as reasonably reliable predictions.

**Figure 9:**
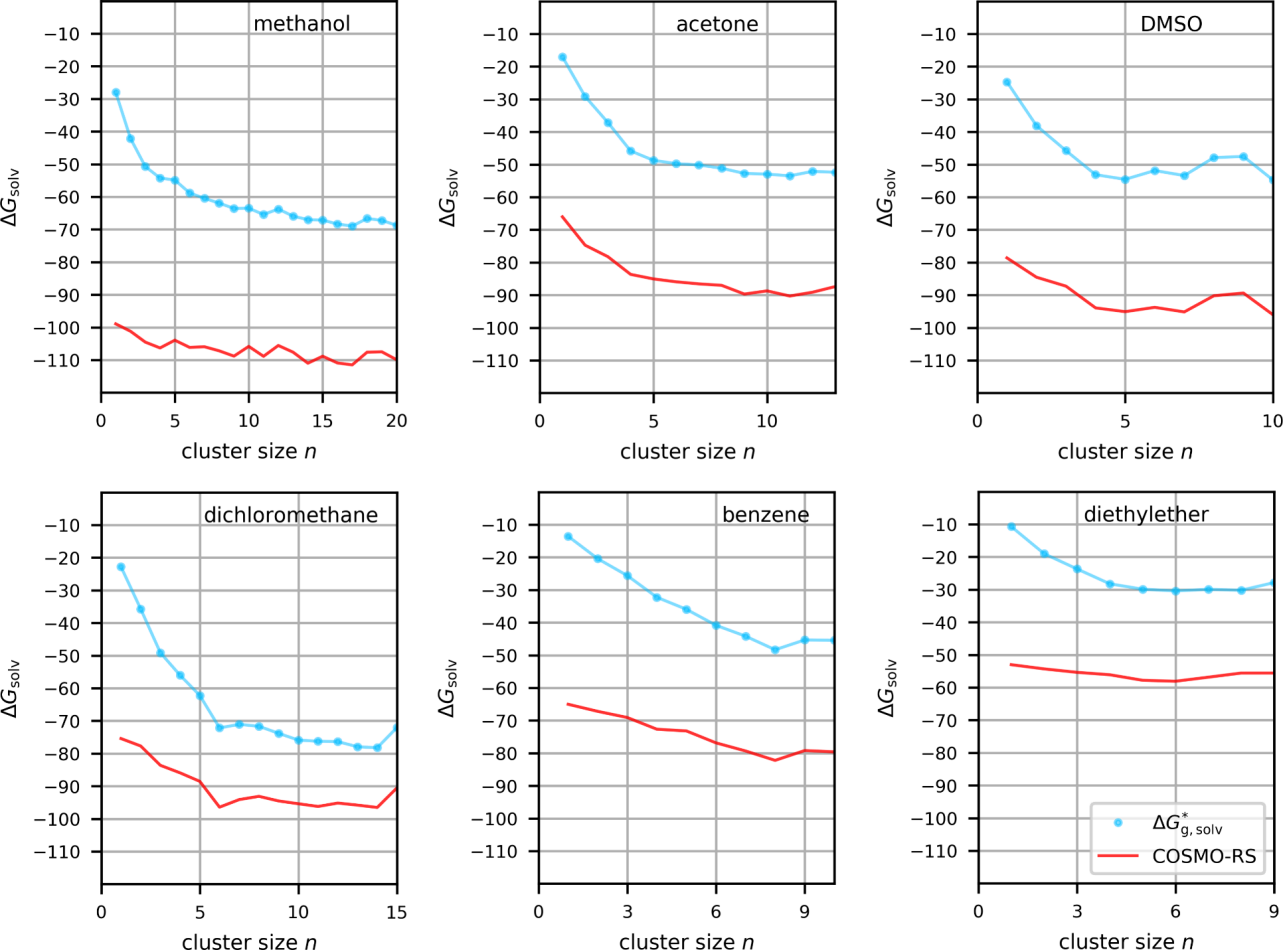
Gibbs free energy of solvation in kcal/mol for F^–^ in different solvents calculated with the cluster-continuum approach for different cluster sizes (with ωB97X-D/def2-TZVPD), either without embedding (blue) or with COSMO-RS embedding (red).

**Table 5:** Averaged Gibbs Free Energy of Solvation $\overset{®}{\Delta G_{solv}^{*}}$ in kcal/mol from Cluster-Continuum Calculations (with *ω*B97X-D/def2-TZVPD and COSMO-RS Embedding) for the Fluoride Ion in Different Solvents^a^

solvent	$$\overset{®}{\Delta G_{solv}^{*}}$$	range of *n*	$\Delta_{n}$ ^a^	$$\Delta G_{solv}^{COSMO‐RS}$$
water	–105.7	7–16	1.3	–106.1
MeCN	–94.1	7–16	1.1	–76.2
MeOH	–107.0	6–13	1.9	–98.9
acetone	–88.1	6–13	2.2	–68.1
DMSO	–94.5	4–7	0.7	–75.7
DCM	–95.6	9–14	1.1	–82.7
benzene	–79.4	6–10	2.8	–61.0
diethyl ether	–56.2	2–9	1.9	–53.8

a Pure COSMO-RS results are also included.

b Maximum deviation from the average within the range of *n* (in kcal/mol).

Relatively smooth curves are found for all solvents, the convergence for benzene may just have been reached near *n* = 8 (Figure 9). The shorter oscillations for methanol likely reflect its protic nature, while all other solvents in Figure 9 are aprotic ones. However, we note the above discussion of charge-assisted hydrogen bonding to the highly compact fluoride ion even in nominally aprotic solvents like MeCN.^15^ Indeed, the averaged free energies of solvation in at least acetone, DMSO, and DCM are in a similar range as in MeCN (Table 5), indicating such strong charge-assisted hydrogen bonds also in those cases. We note in passing that ab initio MD simulations of the FHF^–^ ion in DCM gave clear indications of similarly strong C–H···F hydrogen bonds.^16^ While the free energies of solvation of fluoride in benzene and diethyl ether are less negative, they still indicate appreciably strong solvation. We note in passing that we computed curves up to different maximal *n* for the different solvents a) due to appreciable computational demands for still larger clusters, e.g., for benzene, and b) due to the fact that some solvent molecules are able to form more than one hydrogen bond to fluoride.

Figure 10 provides structures of low-lying conformers of selected cluster sizes that may be viewed as featuring saturated first solvation shells in different solvents. In MeOH, the first solvation shell seems to be completed at *n* = 5, and a region with small oscillations follows over a larger range of *n* (cf. Figure 9). The *n* = 10 cluster shown indeed features five hydrogen bonds to fluoride in a trigonal bipyramidal coordination (Figure 10), but square pyramidal structures also occur. For acetone, the first solvation shell seems to be completed at *n* = 5, which corresponds to nine hydrogen bonds, as four out of five solvent molecules make two (chelating) hydrogen bonds to fluoride. For more acetone molecules, space around fluoride becomes too crowded, and the number of hydrogen bonds actually *decreases*, e.g., to seven at *n* = 12. DMSO is also able to form such appreciably strong C–H···F hydrogen bonds to fluoride. The QCG procedure suggests the first solvation shell to become completed at *n* = 5 with overall 9–10 hydrogen bonds. As for acetone, the molecules make chelating hydrogen bonds, a shorter one in the range 2.01–2.09 Å and a longer one in the range 2.19–2.34 Å (Table S6 in the Supporting Information). For DCM, up to eight molecules can be found with C–H···F hydrogen bonds, but seven hydrogen bonds appear more stable and frequent, with a range of 1.86–2.28 Å (Table S6). For *n* = 6 we obtain a particularly compact first shell with distances around 1.82 Å (Figure 10). This may explain the dip in the free-energy curve at *n* = 6 (Figure 9). Larger clusters than *n* = 8 typically exhibit seven hydrogen bonds to fluoride. The range *n* = 6–14 leads to a well-converged value for the solvation free energy. For all three cases, acetone, DMSO, and DCM, the original GFN2-xTB-based conformational sampling does not provide a good starting point. Changing to sampling using a force-field level provided better starting points for subsequent DFT refinement (see Computational Details).

**Figure 10:**
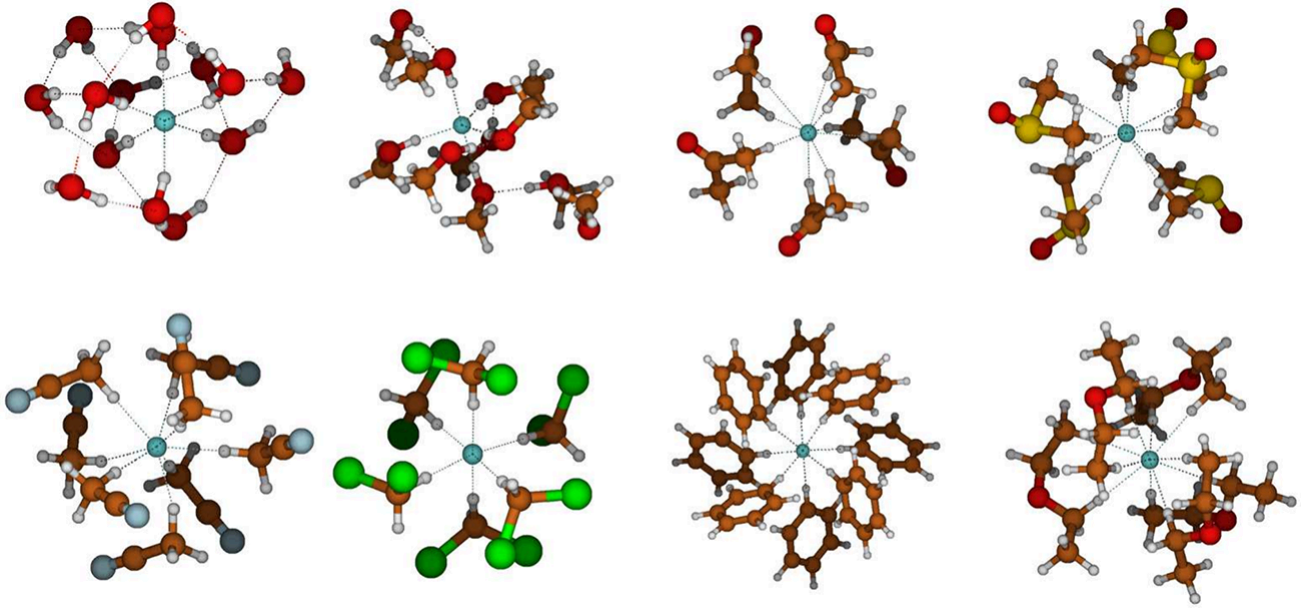
Structure of clusters of F^–^ with different solvent molecules optimized at the BP86-D3­(BJ)/def2-TZVP level (*n* = 14 for water, *n* = 10 for methanol, *n* = 5 for acetone, *n* = 5 for DMSO, *n* = 8 for acetonitrile, *n* = 6 for dichloromethane, *n* = 8 for benzene, and *n* = 5 for diethyl ether).

Even benzene, which does not exhibit electronegative substituents that could strengthen the hydrogen-bond donor character but has somewhat more acidic sp^2^-hybridized C–H units, shows notable C–H···F hydrogen bonds to fluoride, with up to eight molecules fitting into the first solvation shell (with one hydrogen bond each), see Figure 10. At least *n* = 7 is needed to be close to free-energy convergence, generating relatively costly computations. Diethyl ether is interesting due to its flexible internal degrees of freedom. This facilitates conversion between different conformers during optimization. Up to five molecules can fit into the first solvation shell around fluoride, all binding in a chelate-type mode, i.e., with overall ten hydrogen bonds (Figure 10). One hydrogen bond is from a methylene group, the other from a methyl group on the opposite side (Table S6). This seems to provide the least strained coordination. The chelating mode may also explain the shallow dip in the free-energy curve near *n* = 5, 6 (Figure 9). The free energy of solvation in diethyl ether is the lowest of the solvents studied here, due to its sp^3^ C–H bonds and lack of dielectric polarization and of strongly electron-withdrawing substituents. The appreciable free energy for solvation of fluoride in benzene may explain why simple alkali fluorides indeed show finite solubility in benzene,^129^ and why fluorides combined with larger counterions in fact dissolve reasonably well in benzene or ethers and can be used for reactions.^130^

## 5 Conclusions

Application of cluster-continuum solvent models to compute the free energies of solvation of ions has an appreciable history. Here we have suggested a cluster-continuum model work-flow (Figure 3) based on a) automatic conformational sampling at cheap tight-binding or force-field levels, e.g., using the quantum cluster growth algorithm, b) reoptimization of structures and computation of vibrational contributions to free energies at a relatively inexpensive DFT level (BP86-D3­(BJ)/def2-TZVP), c) computation of final electronic energies with higher-level DFT methods (ωB97X-D/def2-TZVPD or DSD-PBEP86/def2-TZVPD), d) embedding in a suitable implicit solvent model (in particular COSMO-RS), and e) selection of conformers with the lowest overall free energy. This procedure has allowed us to go to relatively large cluster sizes and to screen the convergence of computed solvation free energies with the number of explicit solvent molecules for a wide variety of ions in different solvents, while having a good chance of finding low-lying conformers close to the global minimum for not too large cluster sizes. Averaging computed free energies of solvation over a region of cluster sizes with only small oscillations provides well-converged final values. The influences of electronic-structure level and of embedding model (COSMO, SMD, different parametrizations of COSMO-RS) have been evaluated. A good selection of one conformer with low free energy provides stable results, conformational averaging then does not seem to be required.

Application of this workflow to ions in aqueous solution has produced a single-ion scale of Gibbs free energies of hydration closely aligned with a widely accepted scale based on the cluster pair approximation (CPA), to within the combined error margins of both scales of a few kcal/mol. Notably, however, while the CPA scale is anchored to the proton value and then uses differences between free energies of solvation between different ions, the present cluster-continuum values are all fixed individually. We have applied the same cluster-continuum procedure to the individual ${{\mathrm{AsPh}}_{4}}^{+}$ and ${{\mathrm{BPh}}_{4}}^{-}$ ions underlying the basic assumption of equal solvation of large spherical cations and anions of the widely used TATB single-ion solvation scale. These calculations clearly suggest the anion to be more strongly solvated, and the TATB scale has to be corrected. This moves it even further away from the values of the CPA scale. The multiple anchor points of the present cluster-continuum-based scale are seen to provide a robust check on such scales in general.

We also computed single-ion free energies of solvation in MeCN, where we find significant deviations of the cluster-continuum values from a suggested CPA-based scale by about −8 to −10 kcal/mol for anions. Here the suggested CPA-based proton value of −260.2 kcal/mol appears to be less settled, and use of solvent transfer values suggests that the cluster-continuum-based scale reported here may be better founded. This will require further study.

We also provide a detailed computational comparison of the solvation of the fluoride ion in different protic and formally aprotic solvents. While the protic solvents water and methanol exhibit the most negative free energies of solvation, many of the formally aprotic solvents are not far behind. They all exhibit strong, charge-assisted C–H···F hydrogen bonds to the highly compact fluoride ion, often with chelate-type multiple hydrogen bonds from one solvent molecule, e.g., for DCM, DMSO, acetone, or even for diethyl ether. Benzene also shows remarkably strong hydrogen bonding to fluoride, explaining a number of experimental observations.

## Supplementary Material





## Notes

Cartesian coordinates of many DFT-optimized neutral and ionic clusters (ZIP)
Additional six tables and ten figures of solvent properties, free-energy curves, averaged free energies, results with implicit solvent models, effects of conformational averaging, solvation free energies of ion pairs, and structure parameters of ion–solvent clusters (PDF)
