# Supplementary material for: Systematic Evaluation of a Cluster-Continuum-Model Workflow to Compute the Free Energies of Solvation of Ions in Different Solvents
Source: J Phys Chem A. 2026 Apr 28;130(18):3726–42. doi: 10.1021/acs.jpca.6c00886 (PMC13158995; doi:10.1021/acs.jpca.6c00886)
Supplement: Supplementary file 2 [file jp6c00886_si_002.pdf]

Supporting Information:

Systematic evaluation of a cluster-continuum-model workflow to compute the free energies of solvation of ions in different solvents

Morten Lehmann, Froze Jameel and Martin Kaupp*

Technische Universität Berlin, Institut für Chemie, D-10623 Berlin, Germany

E-Mail: martin.kaupp@tu-berlin.de

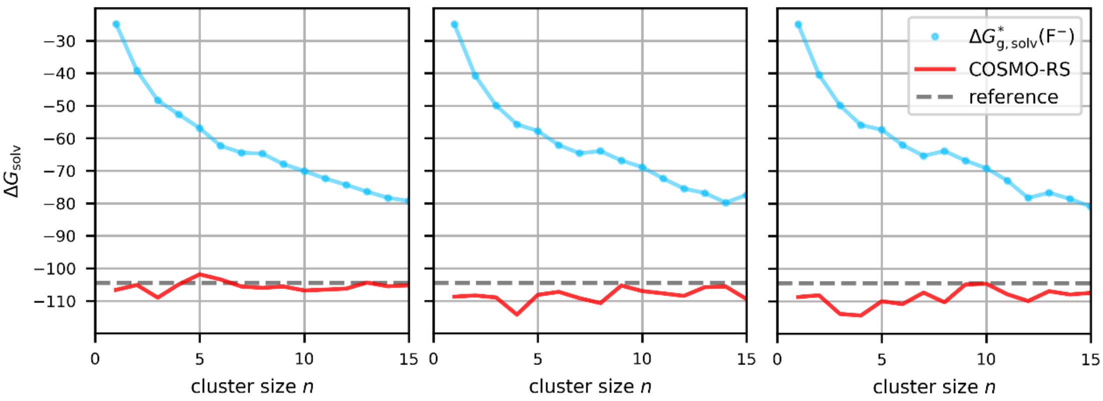

Figure S1: Gibbs free energy of solvation in kcal/mol of F^- in water calculated with the cluster continuum approach (ωB97X-D/def2-TZVPD) for different cluster sizes. The dashed horizontal line corresponds to the CPA-based reference value. The DFT structure optimizations were performed without COSMO (left) and with COSMO (middle and right). The initial construction of the clusters with QCG employed no implicit solvation model (left and middle) or the ALPB (analytical linearized Poisson-Boltzmann) model (right). The corresponding results in kcal/mol averaged over a range of 10 to 15 are −105.8 (left), −107.0 (middle), and −107.1 (right).

Table S1: Density ρ in g/cm³ and dielectric constants ε of the solvents at 298.15 K.

Solvent	ρ[a]	ε(COSMO)[a]	ε(SMD)
Water	0.997	78.4	78.355
Acetonitrile	0.777[b]	35.7	35.688
Methanol	0.786[b]	32.6	32.613
Acetone	0.785[b]	---	---
Dimethyl sulfoxide	1.101	---	---
Dichloromethane	1.313[b]	---	---
Benzene	0.873[b]	---	---
Diethylether	0.708[b]	---	---

[a] Taken from Ref. [1]. We note that the COSMO-RS calculations are based on a COSMO run with infinite dielectric constant. [b] Density was taken as the average of densities at 293.15 K and 303.15 K.

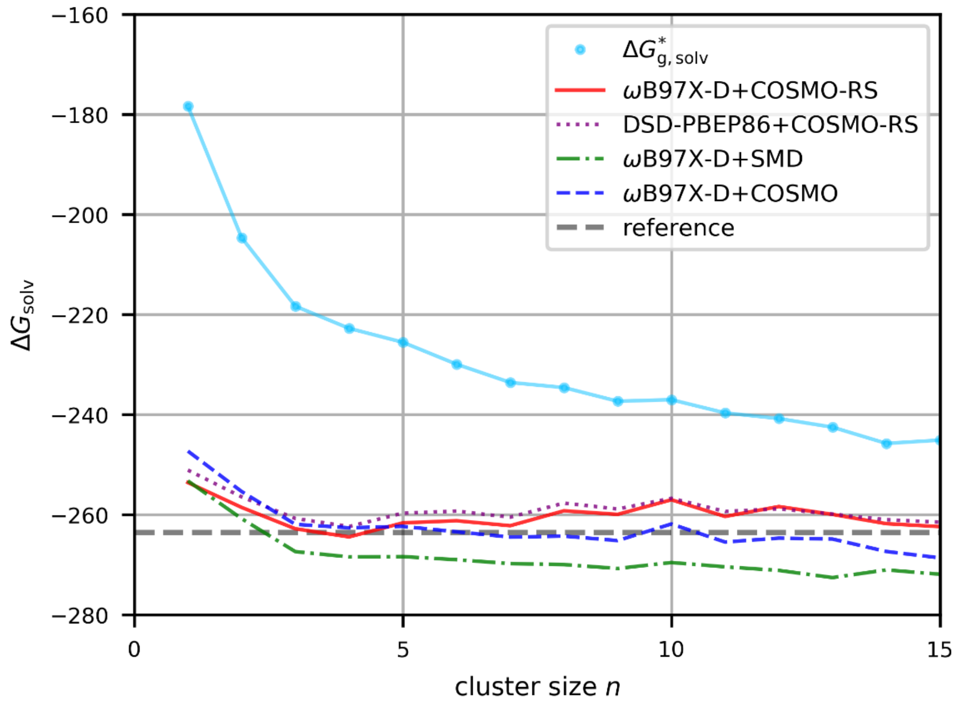

Figure S2: Gibbs free energy of solvation in kcal/mol of H^+ in methanol calculated with the cluster-continuum approach for different cluster sizes, different embeddings and different computational levels for the electronic energies. Compared to the CPA-based reference value (dashed line).

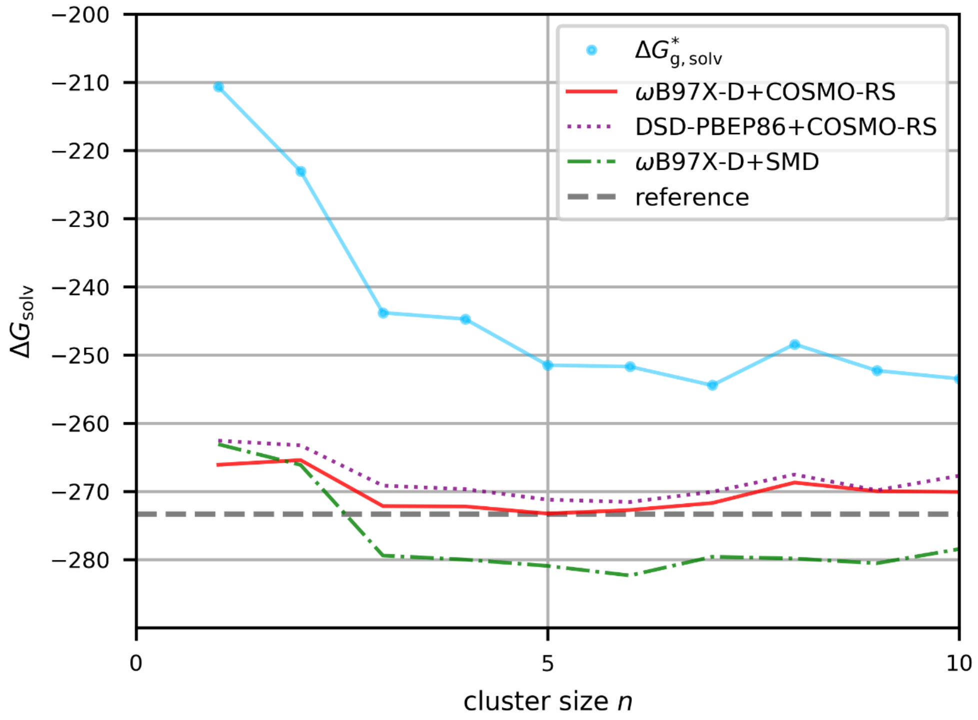

Figure S3: Gibbs free energy of solvation in kcal/mol of H^+ in DMSO calculated with the cluster-continuum approach for different cluster sizes, different embeddings and different computational levels for the electronic energies. Compared to the CPA-based reference value (dashed line).

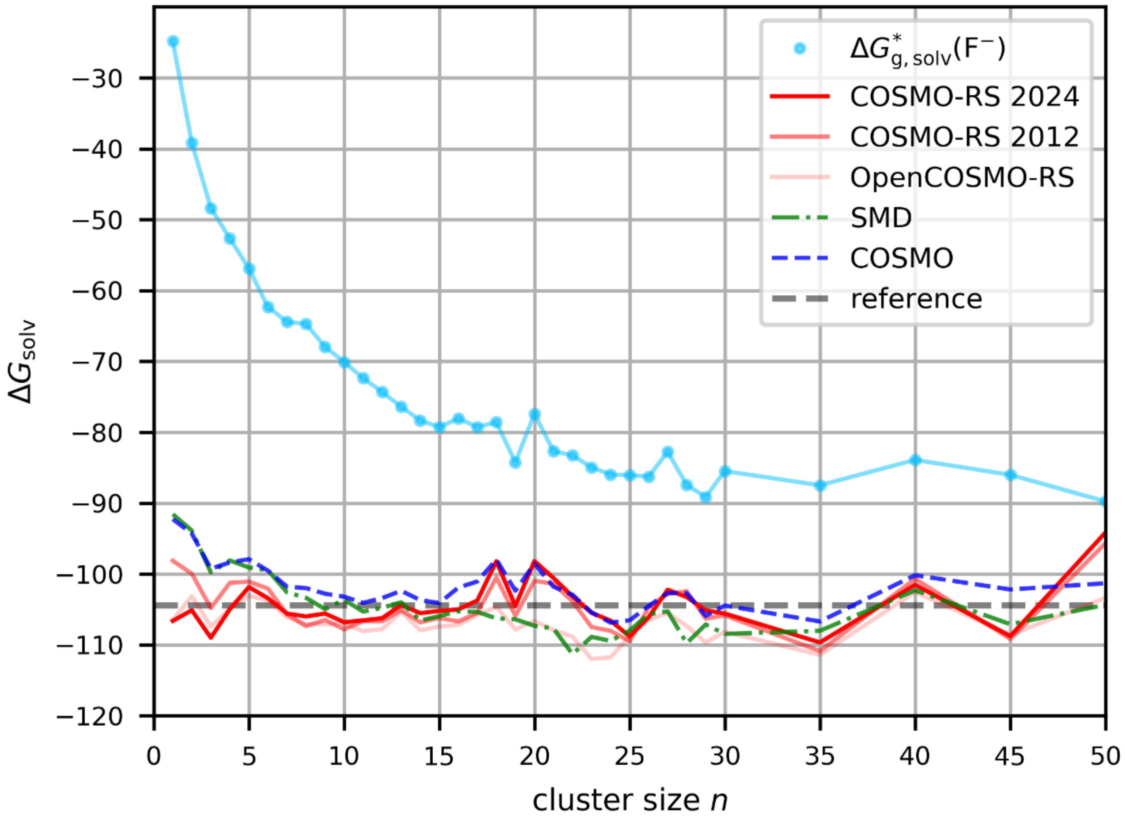

Figure S4: Gibbs free energy of solvation in kcal/mol of F^- in water calculated with the cluster-continuum approach for different cluster sizes and different embeddings, using generally the conformer with the overall lowest free energy. Compared to the CPA-based reference value (dashed line).

Table S2: Comparison of $\overline{\Delta G^{*}_{\mathrm{solv}}}$ in water from cluster-continuum calculations with COSMO-RS embedding for different XC-functionals (ωB97X-D and in parentheses DSD-PBEP86) with results from pure implicit solvent models and the CPA-based reference values in kcal/mol.

	$\overline{\Delta G^{*}_{\mathrm{solv}}}$ [a]	$\Delta G^{\mathrm{SMD}}_{\mathrm{solv}}$	$\Delta G^{\mathrm{COSMO}}_{\mathrm{solv}}$	$\Delta G^{\mathrm{COSMO-RS}}_{\mathrm{solv}}$	Ref.[2]
F^-	−105.7 (−103.8)	−87.1	−88.0	−106.1	−104.4
Cl^-	−74.3	−65.4	−71.5	−75.6	−74.5
Br^-	−67.9	−53.3	−66.9	−75.8	−68.3
I^-	−59.0	−62.2	−60.6	−70.2	−59.9
Li^+	−125.1 (−129.4)	−90.1	−103.9	−97.3	−128.4
Na^+	−100.4 (−106.7)	−72.2	−90.7	−87.8	−103.2
K^+	−79.8 (−83.9)	−59.6	−73.5	−71.5	−86.0
NH_4^+	−83.2	−82.0	−82.4	−82.7	−85.2

[a]Following the procedure explained in the main text, see also Table 2.

Determination of optimal cluster-size ranges for averaging free energies of solvation within the current cluster-continuum workflow

For all cluster sizes $1 \leq i_0 \leq n_{\max}$ and $i_0 \leq m \leq n_{\max}$ (with $[i_0, m]$ being the range over which the results are averaged), the following free-energy average was determined:

$$\overline{\Delta G_{\mathrm{solv}}}(i_0, m) = \frac{1}{m - i_0} \sum_{i=i_0}^{m} \Delta G_{\mathrm{solv}}(i) \,. \tag{S1}$$

Then, a result can be selected with the largest possible range and the smallest possible deviations from the average, Δ_n, by determining the minimum of

$$X = \frac{\Delta_n}{0.5 \operatorname{erf}\left(0.4 \cdot (m - i_0 - 6)\right) + 0.5} \,. \tag{S2}$$

All Δ_n below $0.5 \frac{\mathrm{kcal}}{\mathrm{mol}}$ were set to $0.5 \frac{\mathrm{kcal}}{\mathrm{mol}}$ to ensure that accidentally small deviations over a very small range do not artificially favor these very small ranges.

There is one exception from this procedure. For the solvation of fluoride in benzene and DMSO, the following modified equation was used:

$$X^* = \frac{\Delta_n}{0.5 \operatorname{erf}\left(0.4 \cdot (m - i_0 - 4)\right) + 0.5} \,. \tag{S3}$$

As there is only a small well-converged range in both cases, Eq. S1 would lead to large ranges with large deviations, including results which are clearly not yet converged (e.g., clusters of size 4 and 5 for fluoride in benzene).

Table S3: Comparison of COSMO-RS results for free energies of solvation of different ions in water in kcal/mol with two different parametrizations.

	BP_TZVPD_FINE_HB2012_C30_1201	BP_TZVPD_FINE_24
F^-	−92.9	−106.1
Cl^-	−76.2	−75.6
Br^-	−71.3	−75.8
I^-	−66.7	−70.2
Li^+	−87.4	−97.3
Na^+	−80.0	−87.8
K^+	−67.4	−71.5

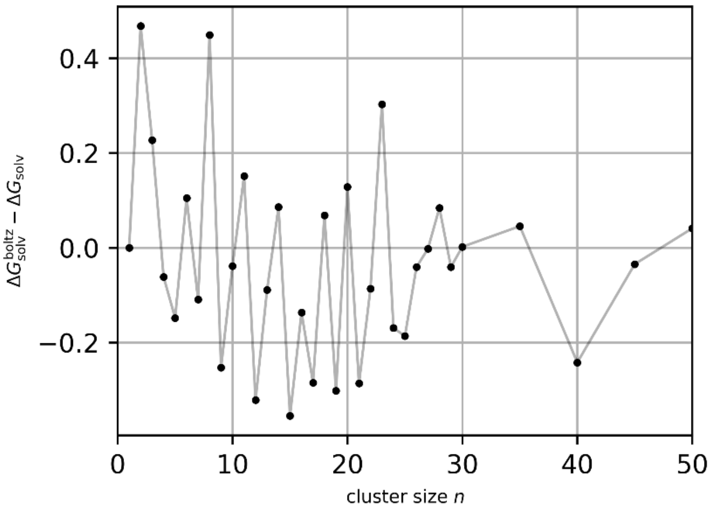

Figure S5: Differences between the Gibbs free energy of solvation in kcal/mol of fluoride in water calculated with the cluster continuum approach when using either the lowest conformer or a Boltzmann average at 298 K at a given cluster size. The Boltzmann-averaged value was calculated from averaged Gibbs free energies $G^{boltz} = \sum_i^N p_i G_i$ with the Gibbs free energy of a conformer G_i out of an ensemble of N conformers and the Boltzmann weights $p_i = \frac{e^{-G_i/k_B T}}{\sum_j^N e^{-G_j/k_B T}}$. ωB97X-D/def2-TZVPD results with COSMO-RS embedding.

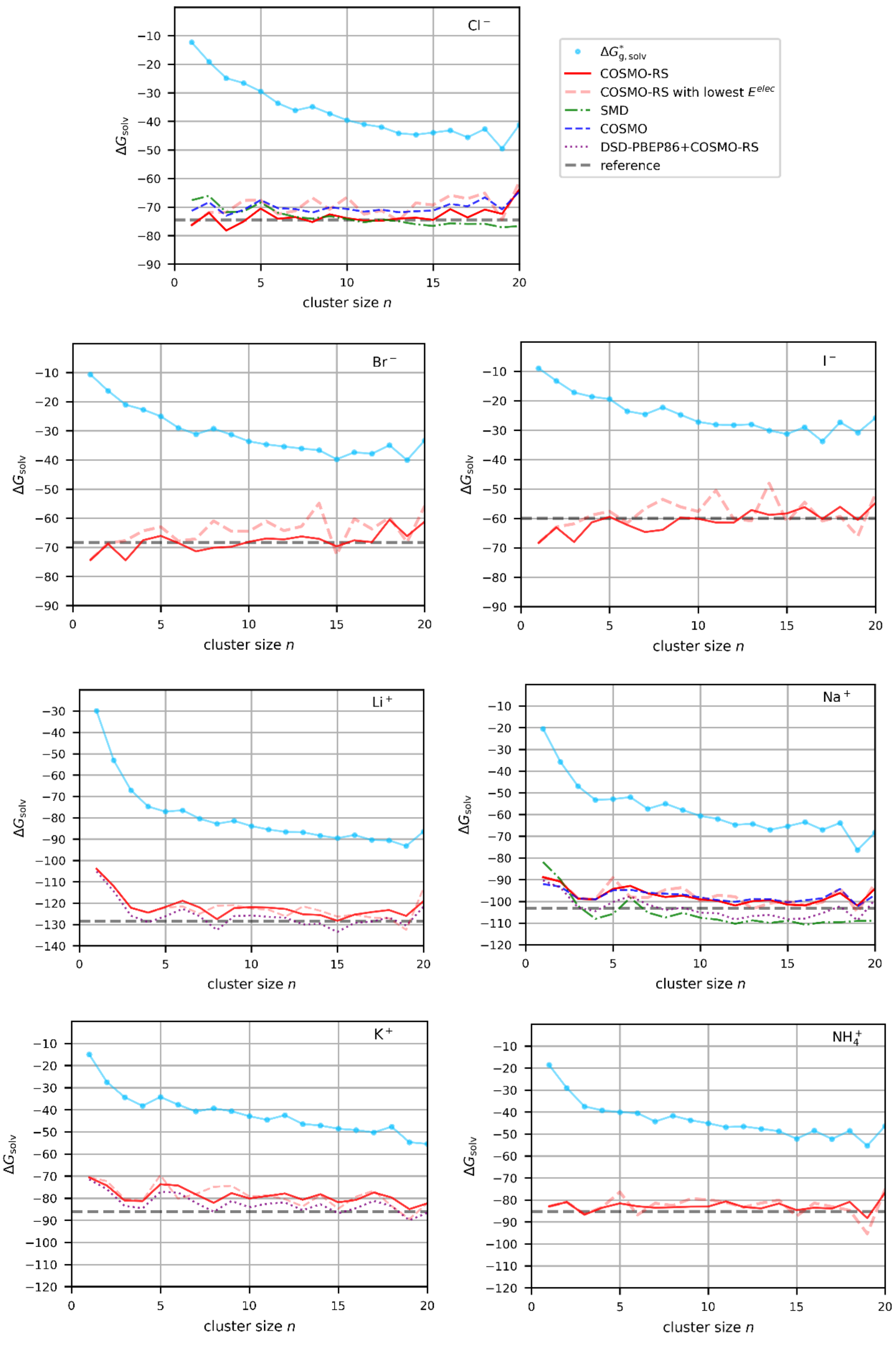

Figure S6: Gibbs free energy of solvation in kcal/mol of different ions in water calculated with the cluster continuum approach for different cluster sizes, with different embeddings or without embedding. The dashed horizontal line corresponds in each case to the CPA-based reference value. ωB97X-D/def2-TZVPD results unless noted otherwise. While most curves are based on the conformer with the lowest free energy, we also show the ωB97X-D/def2-TZVPD+COSMO-RS curve based on the conformer with the lowest electronic energy.

Modified workflow using SOLVATOR/GOAT

For comparison with the QCG-based cluster generation, we also tested in a few cases use of the SOLVATOR module in the ORCA code, version 6.0.0, for the initial generation of the clusters (usually at GFN2-xTB level). A stochastic method was employed to place a predefined number of solvent molecules in a stepwise manner for n = 1−30. Conformational ensembles were generated using the Global Optimizer Algorithm (GOAT).[3] In contrast to the metadynamics-based sampling in CREST, GOAT employs basin hopping, simulated annealing, and tabu search to locate the lowest-lying minimum for a given solute–solvent ensemble while generating molecular conformers along the optimization pathway. The GOAT calculations were run until a lowest-lying minimum was identified, and all conformers within an energy window of 6 kcal/mol were retained to construct the conformational ensemble. If the number of conformers exceeded 30, k-medoids clustering[4] based on RMSDs and relative energies was performed to preserve conformational diversity. From this clustering, 30 representative conformers were selected to describe the conformational space. DFT reoptimization, energy computations, and solvent embedding were subsequently done identically to the QCG-based computations (see main text).

Table S4: Gibbs free energy of solvation $\overline{\Delta G^*_{\text{solv}}}$ in kcal/mol for different ions in water from the cluster-continuum approach (at ωB97X-D/def2-TZVPD+COSMO-RS level) using SOLVATOR/GOAT for the initial construction of the clusters, in comparison with CPA-based reference data. See Table 2 in main text for analogous data based on the QCG approach.

	$\overline{\Delta G^*_{\text{solv}}}$ [a]	Δ_{max} [b]	range of n	Δ_n [c]	Ref.[1]
F^-	−106.3	8.6	6−15	1.8	−104.4
Cl^-	−73.2	14.0	9−17	2.4	−74.5
Br^-	−66.3	21.6	8−15	3.0	−68.3
I^-	−60.3	15.6	9−19	1.4	−59.9
H^+	−268.8	10.1	11−18	0.6	−265.9
Li^+	−123.8	22.8	9−17	1.5	−128.4

[a] Average over a range of cluster sizes n with small oscillations. [b] Maximum deviation in the range n = 1 – 20 from the CPA-based reference value. [c] Maximum deviation from the average in the given range.

Figure S7 provides for comparison cluster-continuum hydration free-energy curves as a function of n for some of the same ions, but now based on the initial conformer generation performed using the SOLVATOR/GOAT scheme in the ORCA code (see Computational Details). As for the QCG-based curves used throughout this work, we see that conformer selection according to the lowest overall free energy provides smoother curves than selection according to the lowest electronic energy. Overall, the resulting curves have somewhat larger oscillations, and we sometimes had to rerun the SOLVATOR calculations to obtain smoother curves. Averaging over a range of n with relatively small oscillations nevertheless still tends to work well. The results in Table S4 should be compared to the corresponding QCG-based averages in Table 2 of the main text. The largest difference (1.8 kcal/mol) is found for Br^-, the other values agree to within better than 1 kcal/mol, i.e., also clearly to within the stated error margins of CPA-based scales. Indeed, for Br^- we see the least smooth curve among the SOLVATOR-based ones (Figure S7).

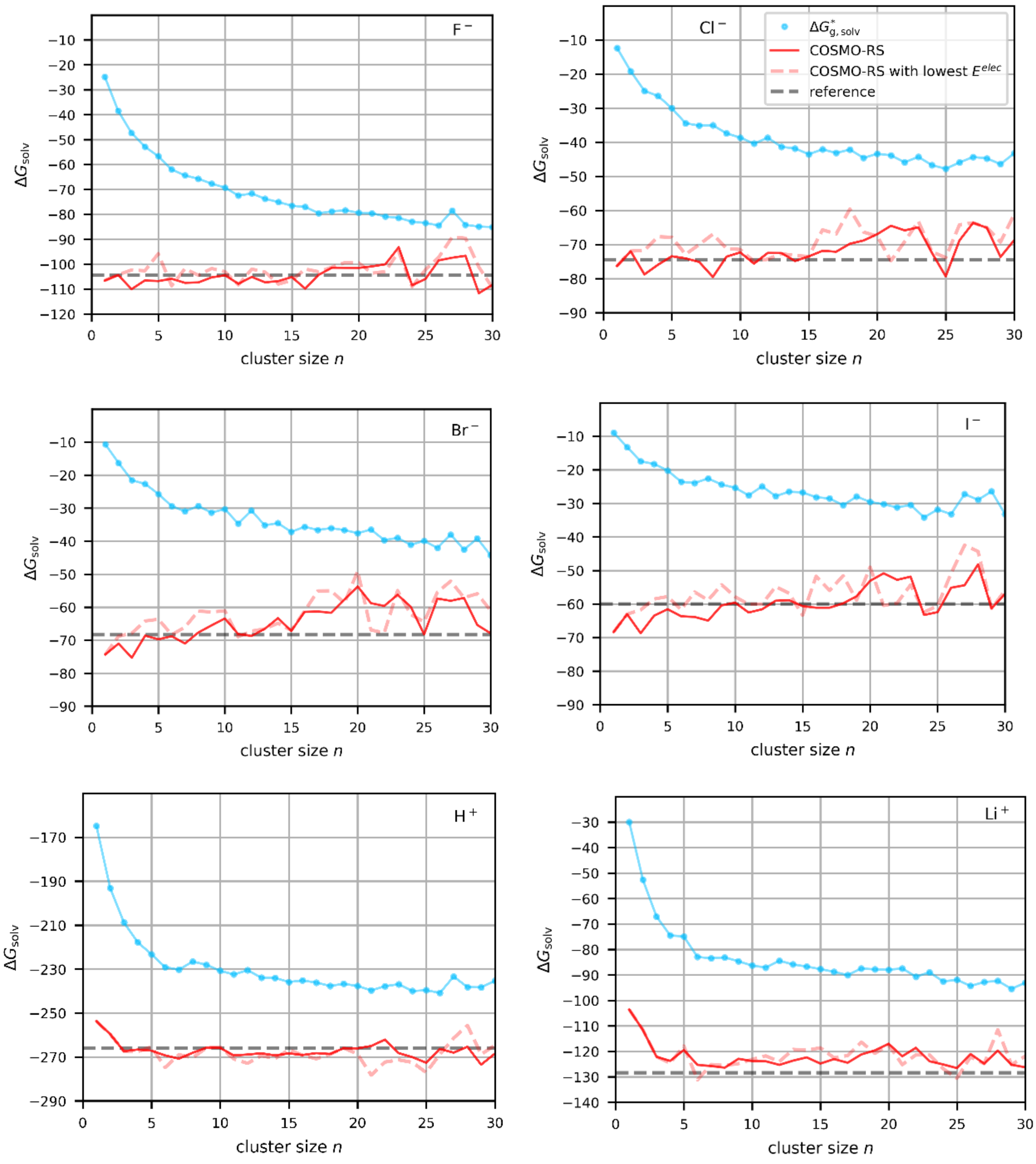

Figure S7: Gibbs free energy of solvation in kcal/mol for various ions in water calculated with the cluster continuum approach (ωB97X-D/def2-TZVPD results with COSMO-RS embedding) for different cluster sizes using SOLVATOR/GOAT for the initial construction of the clusters, but with an otherwise analogous work flow as for the QCG-based calculations shown elsewhere in this work (cf. Figures 4-9, S6).

Table S5: Gibbs free solvation energies of ion pairs in water in kcal/mol calculated as the sum of the averaged cluster-continuum results (ωB97X-D/def2-TZVPD level) with ($\overline{\Delta G^{*}_{\mathrm{solv}}}$) and without ($\overline{\Delta G^{*}_{\mathrm{g,solv}}}$) COSMO-RS embedding.

	$\overline{\Delta G^{*}_{\mathrm{solv}}}(\mathrm{A^+})$	$\overline{\Delta G^{*}_{\mathrm{solv}}}(\mathrm{X^-})$	$\overline{\Delta G^{*}_{\mathrm{solv}}}(\mathrm{AX})$	Tissandier et al.[5]	Δ[a]	$\overline{\Delta G^{*}_{\mathrm{g,solv}}}(\mathrm{A^+})$[b]	$\overline{\Delta G^{*}_{\mathrm{g,solv}}}(\mathrm{X^-})$[b]	$\overline{\Delta G^{*}_{\mathrm{g,solv}}}(\mathrm{AX})$	Δ[a]
HF	−269.0 (9−17)	−105.7 (7−16)	−374.7	−370.3	−4.4	−237.1 (15−20)	−86.4 (24−50)	−323.5	46.8
HCl	−269.0 (9−17)	−74.3 (10−15)	−343.3	−340.5	−2.8	−237.1 (15−20)	−44.4 (14−20)	−281.5	59.0
HBr	−269.0 (9−17)	−67.9 (9−17)	−336.9	−334.1	−2.8	−237.1 (15−20)	−37.2 (15−20)	−274.3	59.8
HI	−269.0 (9−17)	−59.0 (9−18)	−328.0	−325.1	−2.9	−237.1 (15−20)	−30.4 (14−19)	−267.5	57.6
LiF	−125.1 (12−19)	−105.7 (7−16)	−230.8	−232.8	2.0	−89.7 (15−20)	−86.4 (24−50)	−176.1	56.7
LiCl	−125.1 (12−19	−74.3 (10−15)	−199.4	−203.1	3.7	−89.7 (15−20)	−44.4 (14−20)	−134.1	69.0
LiBr	−125.1 (12−19	−67.9 (9−17)	−193.0	−196.7	3.7	−89.7 (15−20)	−37.2 (15−20)	−126.9	69.8
LiI	−125.1 (12−19	−59.0 (9−18)	−184.1	−187.7	3.6	−89.7 (15−20)	−30.4 (14−19)	−120.1	67.6
NaF	−100.4 (10−17)	−105.7 (7−16)	−206.1	−207.6	1.5	−67.4 (14−20)	−86.4 (24−50)	−153.8	53.8
NaCl	−100.4 (10−17)	−74.3 (10−15)	−174.7	−177.8	3.1	−67.4 (14−20)	−44.4 (14−20)	−111.8	66.0
NaBr	−100.4 (10−17)	−67.9 (9−17)	−168.3	−171.4	3.1	−67.4 (14−20)	−37.2 (15−20)	−104.6	66.8
NaI	−100.4 (10−17)	−59.0 (9−18)	−159.4	−162.5	3.1	−67.4 (14−20)	−30.4 (14−19)	−97.8	64.7
KF	−79.8 (8−16)	−105.7 (7−16)	−185.5	−190.4	4.9	−51.0 (15−20)	−86.4 (24−50)	−137.4	53.0
KCl	−79.8 (8−16)	−74.3 (10−15)	−154.1	−160.6	6.5	−51.0 (15−20)	−44.4 (14−20)	−95.4	65.2
KBr	−79.8 (8−16)	−67.9 (9−17)	−147.7	−154.2	6.5	−51.0 (15−20)	−37.2 (15−20)	−88.2	66.0
KI	−79.8 (8−16)	−59.0 (9−18)	−138.8	−145.3	6.5	−51.0 (15−20)	−30.4 (14−19)	−81.4	63.9

[a]Deviation from experiment at the given level. [b]The ranges for these results were determined visually as the results without COSMO-RS embedding are clearly not converged within the available cluster-size ranges.

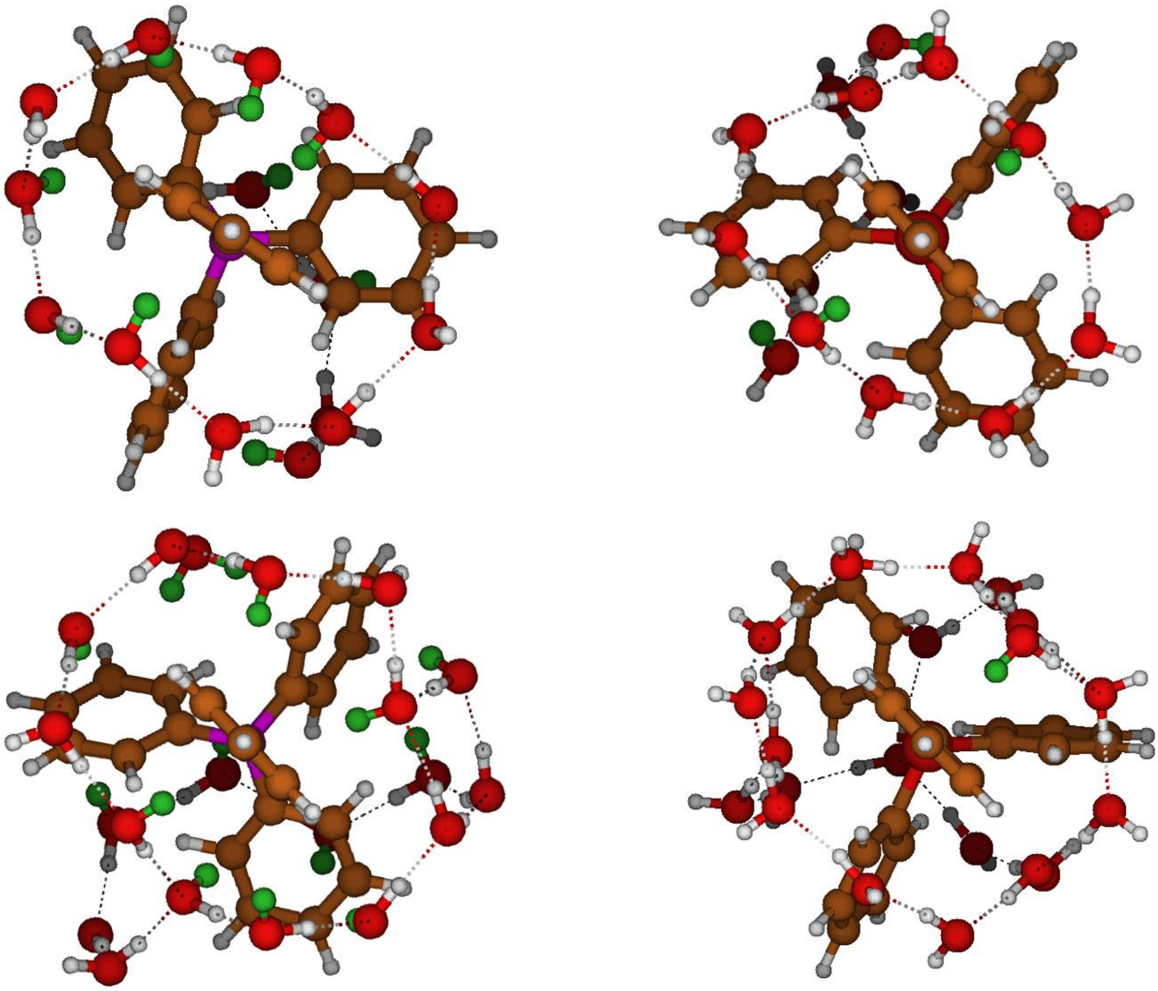

Figure S8: Structure of clusters of BPh_4^- (left) and $AsPh_4^+$ (right) with 15 (top) and 20 (bottom) water molecules optimized at BP86-D3(BJ)/def2-TZVP level. Water hydrogen atoms directed towards the phenyl rings are marked by green color.

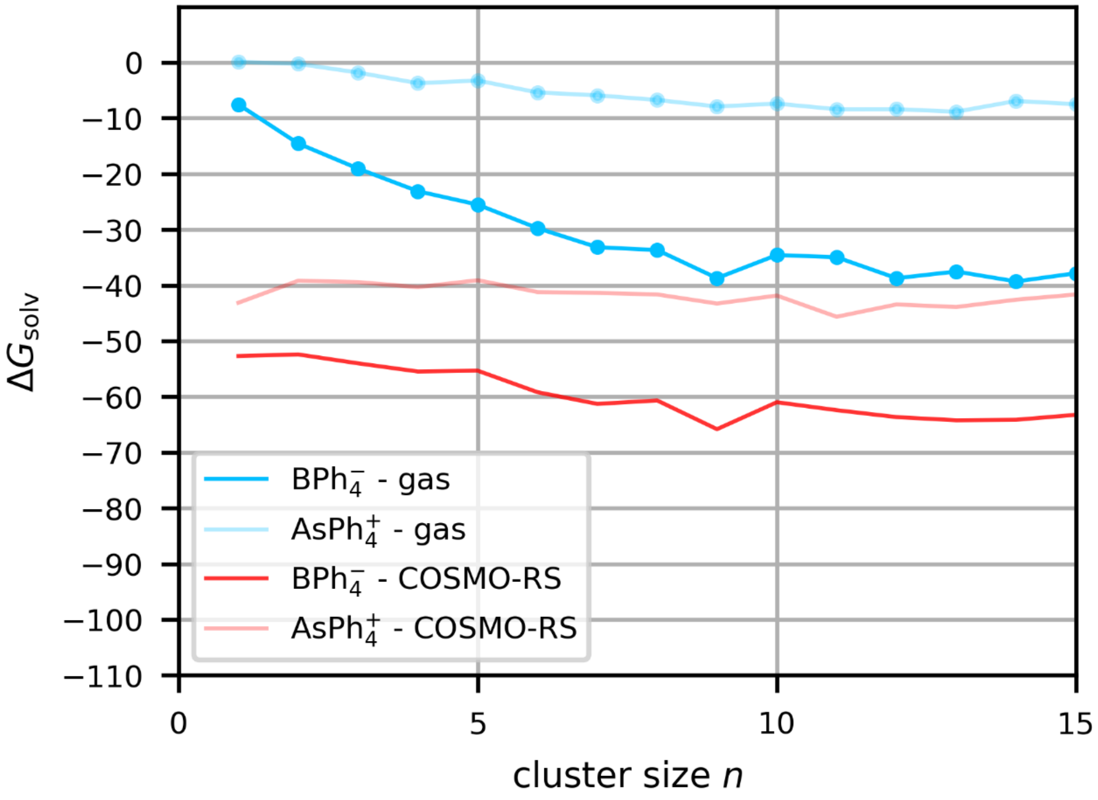

Figure S9: Gibbs free energy of solvation in kcal/mol for BPh_4^- and $AsPh_4^+$ in dichloromethane calculated with the cluster-continuum approach for different cluster sizes at ωB97X-D/def2-TZVP level with or without COSMO-RS embedding.

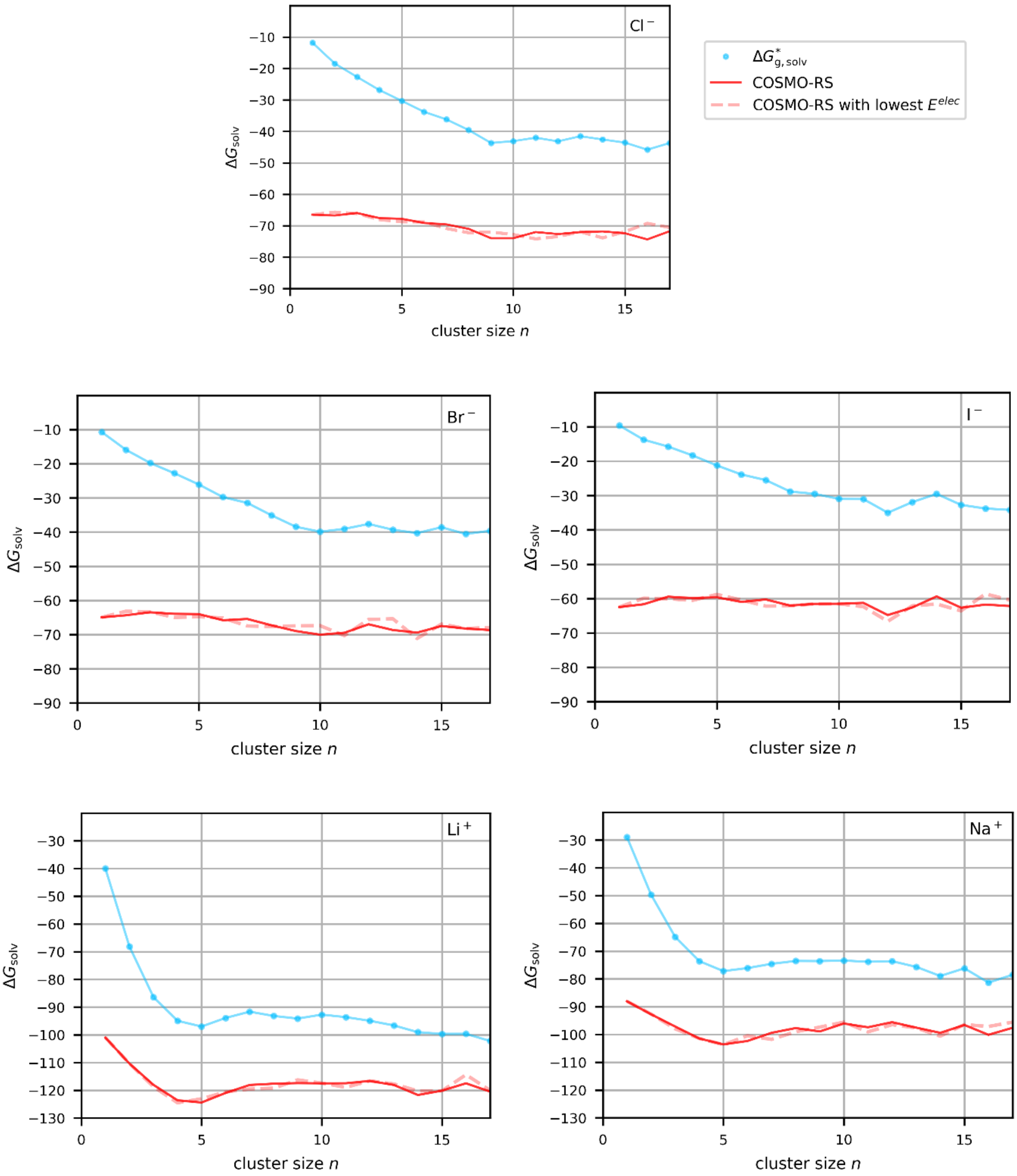

Figure S10: Gibbs free energy of solvation in kcal/mol for different ions in acetonitrile calculated with the cluster-continuum approach for different cluster sizes. ωB97X-D/def2-TZVPD results with or without COSMO-RS embedding, in the former case based either on the conformer with the lowest free energy (red solid line) or lowest electronic energy.

Table S6: Number of hydrogen bonds *m* and closest H···F distances in Å in fluoride-solvent clusters of size *n* optimized at BP86-D3(BJ)/def2-TZVP level for some low-energy conformers.

solvent	*n*	*m* [a]	range of F···H distances
acetonitrile	8	8	1.96−2.08
methanol	6	6	1.62−1.75
acetone	5	9	1.92, 2.06−2.24
DMSO	5	10	2.01−2.09, 2.19−2.34, 2.63
DCM	6	6	1.82−1.82
	7	7	1.86−2.00, 2.28
benzene	8	8	2.00−2.05
diethylether	5	5	2.10−2.28 (CH_2)[b]
		5	2.09−2.21(CH_3)[b]

[a]Number of hydrogen bonds. [b]Distances for methylene or methyl coordination provided separately.

References

[1] *CRC Handbook of Chemistry and Physics, 106th Edition (Internet Version 2025)*; Rumble, J. R., Ed.; CRC Press/Taylor & Francis, Boca Raton, FL.

[2] Kelly, C. P.; Cramer, C. J.; Truhlar, D. G. Aqueous Solvation Free Energies of Ions and Ion-Water Clusters Based on an Accurate Value for the Absolute Aqueous Solvation Free Energy of the Proton. *J. Phys. Chem. B* **2006**, *110*, 16066–16081.

[3] de Souza, B. GOAT: A Global Optimization Algorithm for Molecules and Atomic Clusters. *Angew. Chem, Int. Ed.* **2025**, *64*, e202500393.

[4] *Data Clustering: Algorithms and Applications*; Aggarwal, C. C., Reddy, C. K., Eds.; CRC Press, 2013.

[5] Tissandier, M. D.; Cowen, K. A.; Feng, W. Y.; Gundlach, E.; Cohen, M. H.; Earhart, A. D.; Coe, J. V.; Tuttle, T. R. The Proton's Absolute Aqueous Enthalpy and Gibbs Free Energy of Solvation from Cluster-Ion Solvation Data. *J. Phys. Chem. A* **1998**, *102*, 7787–7794.
